# Bidirectional transcription initiation marks accessible chromatin and is not specific to enhancers

**DOI:** 10.1186/s13059-017-1379-8

**Published:** 2017-12-28

**Authors:** Robert S. Young, Yatendra Kumar, Wendy A. Bickmore, Martin S. Taylor

**Affiliations:** MRC Human Genetics Unit, MRC Institute of Genetics & Molecular Medicine, University of Edinburgh, Crewe Road South, Edinburgh, EH4 2XU UK

**Keywords:** Enhancer, Transcription, Gene regulation, Cap analysis of gene expression, Chromatin modifications, DNase hypersensitivity

## Abstract

**Background:**

Enhancers are modular regulatory elements that are central to the spatial and temporal regulation of gene expression. Bidirectional transcription initiating at enhancers has been proposed to mark active enhancers and as such has been utilized to experimentally identify active enhancers *de novo*.

**Results:**

Here, we show that bidirectional transcription initiation is a pervasive feature of accessible chromatin, including at enhancers, promoters, and other DNase hypersensitive regions not marked with canonical histone modification profiles. Transcription is less predictive for enhancer activity than epigenetic modifications such as H3K4me1 or the accessibility of DNA when measured both in enhancer assays and at endogenous loci. The stability of enhancer initiated transcripts does not influence measures of enhancer activity and we cannot detect evidence of purifying selection on the resulting enhancer RNAs within the human population.

**Conclusions:**

Our results indicate that bidirectional transcription initiation from accessible chromatin is not sufficient for, nor specific to, enhancer activity. Transcription initiating at enhancers may be a frequent by-product of promiscuous RNA polymerase initiation at accessible chromatin and is unlikely to generally play a functional role in enhancer activity.

**Electronic supplementary material:**

The online version of this article (doi:10.1186/s13059-017-1379-8) contains supplementary material, which is available to authorized users.

## Background

Enhancers are modular, regulatory DNA elements that positively drive gene expression at a distance [1]. They are thought to be central to controlling cellular differentiation and developmental gene expression profiles, and mutations disrupting them have been associated with several Mendelian disorders [2, 3]. Widespread bidirectional transcription initiating proximal to enhancers has been observed [4, 5] where the production of these enhancer RNAs (eRNAs) has been demonstrated to mark active enhancers [6] and is correlated with increased expression from nearby, presumptive target promoters [7, 8].

While most existing enhancer discovery methods are based on a characteristic chromatin profile (high H3K4me1 and low H3K4me3) [9], often in conjunction with DNase hypersensitivity [10, 11], the signal of bidirectional transcription initiation has been advocated as a complementary approach [6, 12, 13] and raises the intriguing possibility that enhancer RNAs, or the action of transcription itself, is mechanistically important for enhancer activity. Of candidate enhancers defined solely using RNA-seq evidence in mouse embryos, and subsequently tested using transgenic assays, only 42% were successfully validated [6]. The FANTOM5 consortium used cap analysis of gene expression (CAGE) transcriptome data to define active enhancers and validated 67–74% of their predictions [13]. These validation rates compare to the 75% obtained when enhancers are defined by their chromatin marks alone [14] and are lower than the 87% validation rate for enhancers defined by the binding of histone acetyltransferase p300 [15]. Direct comparison of validation rates between studies based on different discovery thresholds and validation systems is challenging, so it remains to be seen whether epigenetic marks or bidirectional transcription is more specific and accurate in identifying active enhancers.

The production of RNA transcripts initiating at enhancer elements also raises the question of potential functional roles for some of these eRNAs in mediating enhancer activity. siRNA knockdowns of a number of candidate eRNAs have resulted in reduced gene expression [16, 17]. Others have tethered the eRNA molecule to its cognate enhancer and shown that the mature eRNA molecule is required for enhancer activity [18, 19]. Several eRNAs have also been reported to be responsible for RNA polymerase II recruitment at the target promoter [17, 20]. At the human growth hormone gene locus, however, it is only the act of transcription which is correlated with enhancer activity, as the transcribed sequence can be replaced with no effect on resulting gene expression [21, 22]. An analysis of 124 mouse eRNAs detected no evolutionary constraint within their exonic sequences [7], which suggests that these mature transcripts are not generally required for enhancer function. Similarly, many genic transcription start sites are subject to bidirectional initiation, but with the rapid degradation of the non-coding transcript [23]. More recent work has shown that newly evolved transcription start sites are intrinsically bidirectional and that this is a mechanistic feature which alone does not imply biological activity [24], also arguing against a functional role for eRNAs. Despite the convincing evidence for functionality of a handful of eRNAs [25], there is likely a reporting bias against those that do not show an effect, as it is intrinsically difficult to demonstrate an absence of function. The majority of the thousands of eRNAs identified to date have yet to be experimentally interrogated.

In this study, we investigate the specificity of bidirectional transcription for enhancer identification and its importance for enhancer function. We show that the initiation of both stable and unstable bidirectional transcription can frequently be detected at open chromatin regions that exhibit neither chromatin marks characteristic of enhancers nor enhancer activity. While many active enhancers do exhibit bidirectional transcription initiation, this property alone does not define enhancers. Measures of transcription initiation at candidate enhancers correlate less well with their presumptive gene targets than measures of chromatin accessibility at the same sites. Using population genetic approaches that are less perturbed by mutation rate variation than previous interspecies comparisons [7], we confirm that mature eRNAs do not in aggregate show any evidence for purifying selection within the human population, arguing against a sequence-dependent function for these transcripts. We propose that bidirectional transcription is a by-product of an opening of chromatin at all types of regulatory regions, and although indicative of accessible DNA in a transcriptionally active domain, is not sufficient for identifying active enhancers.

## Results

### Transcription initiation is a pervasive feature of accessible chromatin

**Fig. 1:**
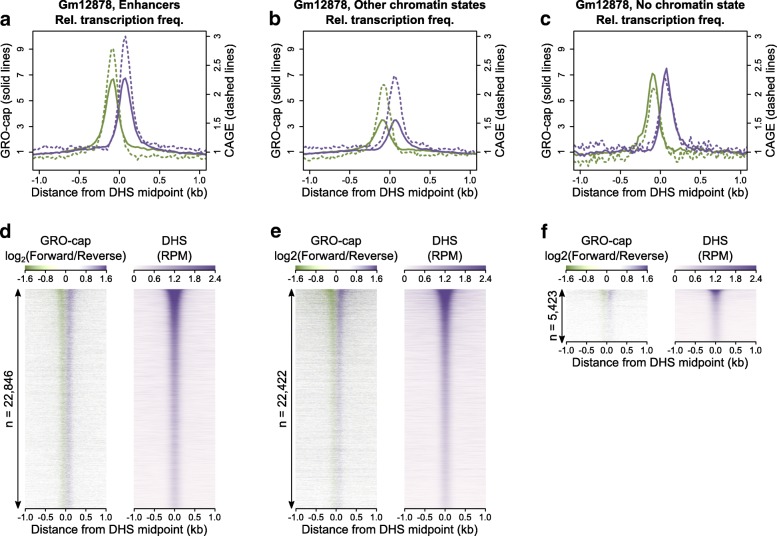
Bidirectional transcription initiates around DHSs but is not a specific mark of active enhancers. **a–c** The fold-change in transcription frequency (fraction of loci with evidence of transcription initiation) for sites with transcription initiation signal in 25-bp consecutive windows around DHS midpoints (x = 0) relative to the mean transcription frequency in the flanking regions: 500 to 1000 bp from the DHS midpoint. Total bidirectional transcription initiation across DHSs in Gm12878 cells as measured by GRO-cap is shown by the *solid lines* while stable bidirectional transcription initiation as measured by CAGE is shown by the *dashed lines*. *Purple lines* consider transcription initiation from the forward strand and *green lines* show transcription initiation from the reverse strand. In all panels, only DHSs that do not overlap annotated promoters were included. **d–f** Heatmaps of GRO-cap signal as measured by log2(Forward/Reverse) RPM and DNase hypersensitivity as measured by RPM around DHS midpoints for the same chromatin state annotations described in **a–c**. *Rows* are ranked by the DNase hypersensitivity signal (RPM). The height of each heatmap corresponds to the total number of DHSs which generated the plot as shown on the y-axis so that shading density is directly comparable between plots

We systematically explored the relationship between chromatin state, transcription initiation, and DNA accessibility by focussing on four well studied cell lines (Gm12878, HepG2, Huvec, and K562), all with (i) detailed maps of chromatin modifications assimilated into chromatin state maps [9], (ii) CAGE-based measures of transcription initiation, and (iii) DNA accessibility as measured by DNase hypersensitivity. Confirming previous studies [4–6, 13], we found CAGE-defined bidirectional initiation of transcription around DNase hypersensitivity site (DHS) midpoints at enhancers. The same pattern of bidirectional transcription initiation was seen at DHSs located in each of the other evaluated chromatin environments and even at DHSs lacking sufficient chromatin modification to be assigned to any chromatin state (Fig. 1; Additional file 1: Figures S1–S4), illustrating that this is not a specific feature of enhancer-associated DHSs. In all chromatin environments the local enrichment of transcription initiation reflects the distribution and extent of DHS signal (Fig. 1; Additional file 1: Figures S1–S4), suggesting a general correspondence between DNA accessibility and availability for transcription initiation.

While CAGE sensitively detects the initiation sites of stable transcripts, unstable transcripts can be under-detected. We identified the initiation of both stable and unstable transcripts using global run-on sequencing enriched for 5′-capped RNAs (GRO-cap) support (Fig. 1), an analysis that was repeated independently using GRO-seq and PRO-seq, which detect both transcription initiation and elongation (Additional file 1: Figures S5–S8). In all chromatin state environments and regions outside chromatin state annotations we consistently found the same pattern of bidirectional transcription initiation from the DHS midpoint (Fig. 1; Additional file 1: Figures S1–S8; Additional file 2: Table S1; Additional file 3: Table S2).

While much bidirectional transcription initiating at enhancers does not produce stable RNA transcripts [25], these results, which are supported by multiple sequencing technologies and cell lines, confirm that higher than background rates of stable and unstable transcription initiation is common to DHSs irrespective of chromatin state annotation. The background noise in these data is low, as indicated by the much reduced frequency of DHSs identified as showing evidence for transcription initiation at over 500 bp from the DHS midpoint (Fig. 1 a–c; Additional file 1: Figures S1–S8a–c) and the clarity of the spatial enrichment in transcription initiation at individual DHSs evident in the heatmaps (Fig. 1d–f; Additional file 1: Figures S1–S8d–f).

Across these genomic contexts, the frequency of transcription initiation approximately corresponds with the level of DNA accessibility, as measured by the strength of the DHS signal (Fig. 1d–f; Additional file 1: Figures S1–S8d–f; Additional file 1: Figures S9 and S10). It suggests that either the presence of accessible chromatin facilitates transcription initiation or, perhaps, that the act of transcription may itself be responsible for driving an increased chromatin accessibility. As this pattern is not specific to any class of DHS studied here, we conclude that neither stable nor unstable bidirectional transcription initiation represent a specific mark for identifying active regulatory elements that function as enhancers.

Transcription initiation does not necessarily extend into productive elongation [23]. To address this we explored the processive efficiency of transcripts initiating at DHSs from each of our chromatin state annotations, comparing them to annotated promoters for protein-coding and long intergenic noncoding RNA (lincRNA) genes (Additional file 1: Figure S11). We found that protein-coding and lincRNA promoters consistently exhibit productive transcription up to and including 250 bp distant from the transcription initiation site, both in the level of extending transcript detected by GRO-seq and in the fraction of transcripts detected. In contrast, transcription initiating at DHSs marked as enhancers, other chromatin states and those DHSs outside chromatin state annotations all behaved similarly, with evidence of transcription decaying rapidly within the first 100 bp from the CAGE-defined transcription initiation site. This points to transcription initiating at all classes of DHS outside of genic and lincRNA promoters being rarely processive and typically extending only for tens of nucleotides before transcription termination.

### Transcription initiation but not transcript stability is associated with activity level at chromatin marked enhancers

**Fig. 2:**
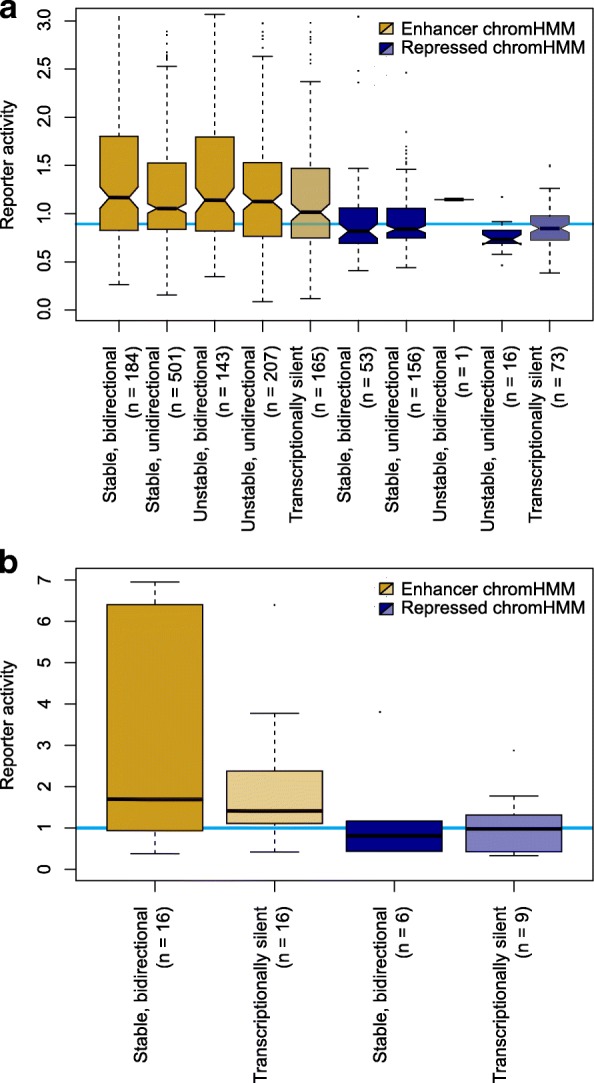
Chromatin-defined enhancer marks rather than transcription are indicative of enhancer activity. **a** Reporter activities for chromatin mark-defined enhancer and repressed regions in K562 cells with stable and unstable bidirectional and unidirectional transcription initiation, and those with no evidence for transcription. The *horizontal blue line* indicates the median reporter activity for all scrambled control sequence assays in K562 cells. **b** As for **a**, in HepG2 cells but only considering transcribed regions to be those with stable, bidirectional transcription initiation. The *blue line* indicates the reporter activity for the transfected empty vector

To further explore the relationship between transcription initiation and enhancer activity we intersected data from 1499 high-throughput enhancer reporter assays of candidate regulatory elements in K562 cells [26] with measures of transcription initiation at their endogenous genomic loci. Sites of unstable transcription initiation were identified as those which lacked CAGE support but displayed evidence of transcription using complementary (GRO-cap, GRO-seq, PRO-seq) technologies designed to identify regions of active transcription initiation [27]. In line with previous reports [6, 13], transcribed enhancers showed significantly higher reporter activity than chromatin-defined enhancers without any detected transcription initiation (Fig. 2; median increased activity 1.1-fold, Mann-Whitney *p* = 0.01). Enhancers producing stable transcripts were not significantly more active in the reporter assays than those producing only unstable transcripts (Mann-Whitney *p* = 0.95), suggesting that neither transcript stability nor the mature transcripts themselves are generally required for enhancer activity. That our classification of transcribed enhancers only required the support of a single CAGE or GRO-cap read, yet still showed a significantly higher activity than those enhancers with no evidence of transcription, confirmed that our choice of cutoff is biologically meaningful.

### Histone modifications rather than transcription are indicative of enhancer activity

Sites with transcription initiation but repressive chromatin marks do not exhibit enhancer activity relative to scrambled controls (median activity 0.9-fold, Mann-Whitney *p* = 0.02), demonstrating that neither bidirectional nor unidirectional initiation of transcription alone predicts enhancer activity (Fig. 2a). In contrast, histone modification-based chromatin state assignments do predict enhancer activity relative to scrambled controls (median activity 1.2-fold, Mann-Whitney *p* < 2.2 × 10^−16^).

To further test our observation that bidirectional transcriptional initiation from accessible chromatin is not specifically associated with enhancer activity we carried out 47 additional reporter assays in HepG2 cells (Fig. 2b). These experiments were performed on enhancer regions specific to HepG2 cells, which are therefore not present in our above analyses of K562 enhancers. Again these results showed that chromatin marks effectively discriminate enhancers from repressed regions (median 1.7-fold greater reporter activity at all chromatin-defined enhancers relative to repressed regions, Mann-Whitney *p* = 0.01) and that bidirectional transcription initiation does not seem to be predictive of enhancer activity as measured by reporter gene output (Fig. 2b; Mann-Whitney *p* ≥ 0.47).

### Open chromatin but not enhancer transcription is a good predictor of proximal gene transcription

**Fig. 3:**
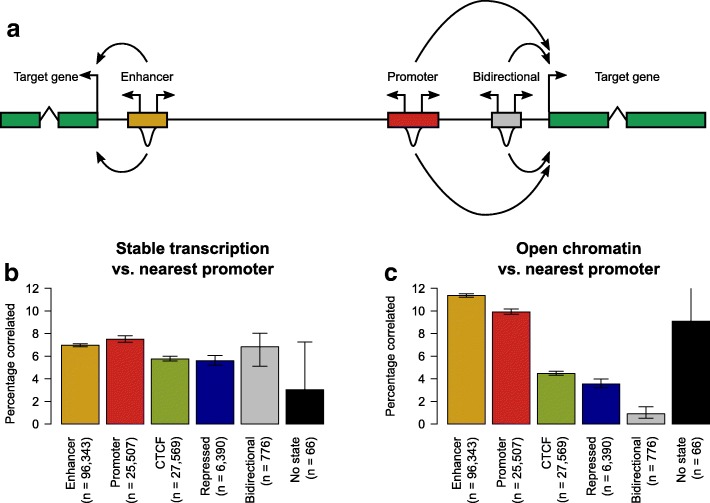
Stable transcription is not indicative of enhancer activity. **a** As shown by the *curved arrows*, the putative target of each chromatin state locus and bidirectionally transcribed-defined enhancer is defined as the nearest annotated gene (shown in the *green boxes*). The activity of each locus as measured by either the level of transcription initiation (the *bidirectional arrows* above each regulatory region) or the strength of the DHS signal (the *peaks* below each regulatory region) is then correlated with transcription initiation at the putative target gene promoter. **b** The percentage of chromatin state loci and bidirectionally transcribed-defined enhancers whose measure of stable transcription initiation is significantly correlated with transcription initiation from the nearest annotated gene promoter. The error bars represent the 95% confidence interval from 1000 samplings of the data with replacement, while the numbers below each bar denote the number of loci tested for a significant correlation. **c** As for **b**, but the correlations being considered are between the level of DHS signal of chromatin state loci and bidirectionally transcribed-defined enhancers and transcription initiation from the nearest annotated gene promoter

As enhancers are defined by their ability to positively drive gene expression in *cis* [1], we next investigated the correlation between proposed markers of enhancer activity and transcription initiation from nearby annotated genic promoters. Our correlations were carried out using data across the four well studied cell lines studied above for which matched chromatin state map, DHS, and CAGE data were all available. To avoid the confounding influence of overlapping gene transcription we only considered regulatory sites that were not contained within the extent of annotated genes nor within 1 kb of their boundaries (Fig. 3a). We found that regardless of chromatin state, typically 6 to 7% of candidate regulatory elements showed (nominally significant) positively correlated transcription initiation with transcription initiation at the nearest annotated genic promoter (Fig. 3b). Chromatin-defined enhancers do not show a markedly increased frequency of correlation relative to CTCF-binding regions or sites with repressive chromatin marks and were modestly less correlated than intergenic sites that exhibit chromatin marks characteristic of promoter activity (orphan promoters). If we consider candidate regulatory elements defined as previously advocated [13] solely on the basis of bidirectional transcription initiation from this limited number of cell types (n = 4), we again find the same approximately 7% fraction positively correlated with the presumptive target (Fig. 3b). This result is consistent with previous observations of correlated expression between adjacent transcriptional units [28], regardless of the function of these adjacent sites of transcription initiation, and suggests that this correlation is driven by regional changes in transcriptional activity over a locus rather than defining the activity of discrete functional elements such as enhancers.

In stark contrast to transcription-based correlations between regulatory elements and annotated promoters, DNase hypersensitivity measures do show clear discrimination between chromatin states in their correlation with genic transcription (Fig. 3c). DNase hypersensitivity at enhancer-marked regions is better correlated with transcription of the nearest gene than hypersensitivity associated with any of the other chromatin state categories (Fig. 3c). Enhancer hypersensitivity appears to have both greater sensitivity (Additional file 4: Table S3; 10,961/6713 = 63% more sites identified) and specificity (11.4 vs. 7.0%) than enhancer transcription for the identification of regulatory correlation (Fig. 3b, c). These results are robust as to whether genic expression was measured as the highest level of transcription from a single transcriptional start site (TSS; Fig. 3), or as the sum of CAGE tags over all annotated promoters for each protein-coding gene (Additional file 1: Figure S12).

We also repeated this analysis using other approaches to identify putative regulatory element–promoter pairs, as not all enhancers target genomically adjacent promoters [29]. When looking for any correlated relationship between regulatory loci and annotated gene promoters within a 500 kb window, we could detect significant correlations at a much greater frequency (~50%; Additional file 1: Figure S13) but still only DNase hypersensitivity showed a marked increased ability to detect correlations at enhancer-marked regions. A similar pattern was observed when we linked regulatory regions to promoter targets within the same physically interacting domain (Additional file 1: Figure S14), which had been identified previously [30]. Interestingly, for candidate enhancers defined by bidirectional transcription alone, their performance relative to other enhancer definitions is markedly improved when constrained to correlation with promoters in the same physically interacting domain (Additional file 1: Figure S14) and particularly to correlation with the best physically interacting promoter (Additional file 1: Figure S15). It is important to note that bidirectionally defined candidate enhancers represent < 1% of the chromatin state defined number (776/96,343). Therefore, the overwhelming majority of correlations reported here suggest that it is the level of open chromatin (as measured by DHS signal strength) at regulatory sites, and not their transcriptional output, which can best be used to specifically identify enhancers and then associate them with putative promoter target(s).

### No evidence of selection on mature eRNA sequences

**Fig. 4:**
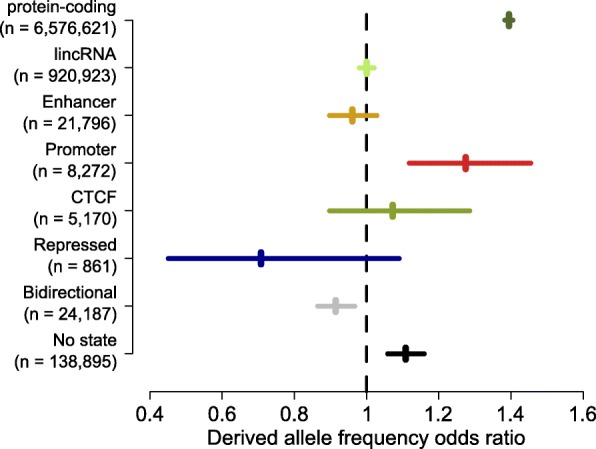
Mature eRNAs do not show signatures of selection within the human population. Odds ratios of frequencies within the deCODE population [49] for rare (<1.5%) and common (>5%) derived alleles compared between exonic and intronic sequences for transcripts overlapping the indicated genome annotations. The numbers of informative SNPs overlapping each category are shown in the *parentheses* next to the axis labels. *Horizontal lines* indicate the 95% confidence interval of the odds ratio estimates. Odds ratios significantly greater than one indicate increased selective constraint in exonic relative to intronic sequence

Having found that the stability of eRNA transcripts does not correspond to measures of enhancer activity (Fig. 2a), we took a complementary approach to test for organism-level biological function in eRNAs by looking for evidence of selective pressures on the DNA sequences encoding these molecules. If the mature eRNA is the functional moiety, we would expect this signal to be concentrated within their exonic, rather than intronic, sequence. It has been previously reported that there is no significant evolutionary constraint within mouse eRNA exons when aligned to the human genome [7]. However, the rapid gain and loss of non-coding regulatory elements through evolution [31, 32] could potentially mask lineage-specific functional constraint when considering deep (between species) sequence comparisons. Fine scale variation in mutation rate could also confound between-species sequence comparisons [33]. Addressing both of these concerns, we measured selective constraint within the human population by comparing the frequency distribution of rare (<1.5%) vs. common (>5%) derived alleles [32] in exonic vs. intronic sequence across various transcript annotation classes (see “Methods”; Additional file 5: Table S4). Purifying selection would be indicated by a relative excess of rare derived alleles in exonic sequence and positive selection indicated by a corresponding increase in the population frequency of derived alleles. As expected, we observed strong purifying selection within protein-coding exons. However, we could detect no evidence of purifying selection in eRNA exons (Fig. 4). Similarly, we did not see evidence of purifying selection in lincRNA exons consistent with previous reports [34]. In contrast to the case for eRNAs and lincRNAs, there is evidence for purifying selection within transcripts initiating proximally to intergenic orphan promoters and, to our surprise, those initiating at DHSs not marked with any chromatin state annotation (Fig. 4). These results suggest that the lack of widespread purifying selection at eRNA exonic sequences between species is also apparent within the human population and again suggests that it is unlikely that the majority of mature eRNA transcripts examined here are biologically functional.

## Discussion

We have shown that low levels of transcription initiation are a common feature of accessible chromatin sites associated with enhancer activity as well as those with other functions (Fig. 1; Additional file 1: Figures S1–S8). Furthermore, increased accessibility of the site (as measured by DNase hypersensitivity) is associated with an increased probability of detecting those transcription initiation sites. Inverting this argument, earlier work has also noted that bidirectional transcription initiation may be a useful proxy for DNase hypersensitivity in diverse environments but only within sites marked with canonical chromatin decorations [35]. These observations are consistent with a model of promiscuous RNA polymerase II transcription initiation on accessible DNA [36] and a major role for chromatin in suppressing spurious transcription initiation [37]. DNA accessibility is not the sole determinant of transcription initiation as colocalization with active regulatory elements within transcription factories [38] or active nuclear compartments [30] and the presence of specific transcription factor transactivation domains can influence transcription output [37, 39]. However, it seems likely that the pervasive, low-level initiation of transcription associated with all categories of highly accessible chromatin represents a form of biological noise rather than specific activity required for the regulation of gene expression. While other approaches to detect enhancers use computational algorithms to remove some of this low-level transcription [13], this also vastly reduces the number of enhancers detected to orders of magnitude less than those detected by chromatin state annotation and thereby discards many genuine, but lowly transcribed, enhancers. We find that the bidirectional initiation of transcription at enhancers is not sufficient to elicit enhancer activity (Fig. 2), nor is bidirectional initiation specific to enhancer activity (Fig. 1).

Despite the lack of specificity for enhancers, measuring bidirectional transcription initiation is certainly not without merit, as it can be used to identify regions of open chromatin in exactly the same sample and source data in which gene expression is quantified [6, 12, 13]. The apparent success of bidirectional transcription alone in defining active enhancers (~70% validation rate [13]) can be explained by the observation that the majority of DNase hypersensitive, and thus transcription-initiating, regions outside of genes are in the context of chromatin-defined enhancers (Additional file 2: Table S1; Additional file 3: Table S2). Transcription initiation provides positive predictive value for accessible, regulatory DNA, but little power to discriminate enhancer from non-enhancer. Further, the measured transcriptional activity of enhancers and other regulatory loci, such as those marked by CTCF or the transcriptionally repressive polycomb complex, are equally correlated with putative target gene expression (Fig. 3). The reduced power to detect correlated enhancer–gene pairs with transcriptome data may in part reflect the reduced coverage of these data at enhancers relative to DNase hypersensitivity.

The pervasive low level initiation of transcription at highly accessible chromatin in diverse contexts suggests the resultant transcripts may be by-products rather than functional entities. There is an important distinction to be made between a molecular measure of activity where there is a detectable molecular species or event and a biological measure of function where the molecular species or event impacts an organism level phenotype. With current technologies we have the power to very sensitively detect the molecular products of the genome (<0.002 copies per cell for the CAGE libraries used in this study [40, 41]), but are all of those products really consequential for the biology of the organism? Our measures of selection tell us that, within the human population, the exonic sequence of transcripts initiating at enhancers is indistinguishable from expectation under neutral evolution (Fig. 4). This does not rule out the possibility that a minority of such sequences are important for organism biology, but overwhelmingly their sequence appears inconsequential for survival or reproductive fitness.

The observation that chromatin marked enhancers work equally well as enhancers whether their associated eRNAs are relatively stable or rapidly degraded (Fig. 2a) supports our measures of selective constraint in suggesting that eRNAs are not generally functionally important products. However, we cannot exclude the possibility that the action of transcription at enhancers (and other DHSs), rather than the resultant transcript, is important for function or maintaining regulation at the site. Indeed, our finding that chromatin marked enhancers without any detected transcription tend to exhibit lower enhancer activity than those with stable or unstable transcription (Fig. 2a) may support this view.

## Conclusions

We have shown that bidirectional transcription can be detected from all types of accessible chromatin, including those regions that have little obvious epigenetic decoration. Furthermore, this transcriptional signal alone is not sufficient to discriminate enhancers from other active regulatory regions in the genome. We propose that bidirectional transcription is predominantly a by-product of an opening of chromatin at all types of regulatory regions and, notwithstanding those published examples of functional eRNAs [25], the majority of the transcripts produced are not likely to be required for regulatory function.

## Methods

### Genome annotation

Protein-coding, miRNA, and lincRNA annotations were extracted from the GENCODE v14 release [42] (June 2012). The promoters for these transcripts were recorded as −300/+100 bp around their annotated TSSs.

Chromatin state maps produced by the SEGWAY algorithm [9] were downloaded for Gm12878, HepG2, Huvec, and K562 cells from the Ensembl Biomart site (release 67, May 2012) [43]. The states ‘Predicted Enhancer’ and ‘Predicted Weak Enhancer/Cis-reg element’ were merged into a single ‘enhancer’ state while the states ‘Predicted Promoter with TSS’ and ‘Predicted Promoter Flank’ were merged into a single ‘promoter’ state. For our cell-specific analyses, the ‘Other’ category includes all chromatin state annotations that do not overlap an ‘enhancer’ state. To further prevent contamination of transcription from enhancers and promoters, those regions within 1 kb of either an enhancer or promoter annotation and annotated as ‘Transcribed’, ‘CTCF’, or ‘Repressed’ were removed from our analyses.

The genomic spans of bidirectionally transcribed-defined enhancers were obtained from http://enhancer.binf.ku.dk/presets/permissive_enhancers.bed [13]. As these enhancer predictions were defined using CAGE libraries from a wide range of cell lines and tissues, we filtered these to include only those loci which showed bidirectional transcription (defined by at least one overlapping CAGE tag on both the forward and reverse DNA strand) in at least one of the four cell types considered here and which would be considered to be an active enhancer in at least one of the cell types by these authors. As for the chromatin state loci, enhancer predictions less than 1 kb from annotated GENCODE [42] or RefSeq [44] gene models were removed before performing the correlation analyses.

For our cross-cell correlation analysis (Fig. 3; Additional file 1: Figures S12–S15), a unified state map was built by merging each state annotation across cell types and then annotating the genome with the merged annotations using the following hierarchy: (1) enhancer, (2) promoter, (3) transcribed, (4) CTCF, (5) repressed. The transcribed regions marked in this manner were not considered in subsequent analyses. In this way, for example, a region is marked as an enhancer if it is annotated as such in at least one of the four cell types but a region is only annotated as repressed if it is marked as repressed in at least one cell type and is also not annotated by any of the other states in any cell type. To further remove any confounding effects of neighbouring gene expression only regions over 1 kb from annotated GENCODE or RefSeq gene models were considered in the correlation analyses. Bidirectionally transcribed enhancers as defined above were also considered in these analyses, without reference to this unified state map.

DHSs for each cell type [45] were obtained directly from the UCSC genome browser (http://hgdownload.cse.ucsc.edu/goldenPath/hg19/encodeDCC/wgEncodeUwDnase/). We downloaded the ‘narrowPeak’ files for each cell type and considered only the intersection of both replicates in our analyses. For our cross-cell correlation analysis, the strength of DNase hypersensitivity for each region was calculated for each cell type as the summed number of reads per kilobase region per million reads mapped (RPKM) measures obtained from both replicates.

### Transcriptome analysis

CAGE data produced by the FANTOM5 consortium [41] were downloaded in BAM format from http://hgdownload.cse.ucsc.edu/goldenPath/hg19/encodeDCC/wgEncodeRikenCage/and all libraries from each cell type were then merged into a single BAM file.

GRO-cap, GRO-seq, and PRO-seq data for K562 and Gm12878 cells [27] were obtained from the GSE60456 series at the Gene Expression Omnibus (GSM1480321, GSM1480323, GSM1480325, GSM1480326, GSM1480237).

Unstably transcribed annotations were first identified as those with transcription initiation supported by GRO-cap evidence and where the genomic extent of overlapping DHSs do not overlap any evidence for stable transcription initiation as measured by CAGE. Subsequently, unstably transcribed regions were similarly identified separately for GRO-seq and PRO-seq evidence. Heat maps for DHS midpoints (±1 kb) were generated using ngsplot v2.61 [46].

Productive elongation was quantified using GRO-seq data in 50-bp windows outwards from the maximally transcribed TSS (as measured by CAGE) within 250 bp of each DHS midpoint. For those DHSs overlapping annotated protein-coding and lincRNA promoters, only CAGE reads which mapped to the annotated strand were considered when measuring elongation rates. For DHSs outside annotated transcribed regions, TSSs were identified separately on each DNA strand.

The expression level for regions annotated across the unified chromatin state map was quantified for each cell type as the RPKM summed across all libraries from that cell and also as the maximum RPKM from an individual TSS’s location for each region. The expression of annotated GENCODE promoters (see above) were quantified in the same way.

Mapped RNA-seq reads from the ENCODE project [47] were downloaded from the UCSC genome browser (http://hgdownload.cse.ucsc.edu/goldenPath/hg19/encodeDCC/wgEncodeCshlLongRnaSeq/) and individual sequencing runs were then asembled into transcripts using Cufflinks v2.2.1 [48], where the GENCODE v14 gene models were supplied as a guide reference annotation (option –g). All other parameters were left at their default values. All transcripts from all cell types were merged into a single set using the Cuffcompare v2.2.1 program. Transcript expression was quantified across cell types and subcellular fractions as the number of fragments per kilobase per million reads mapped (FPKM) using Cuffdiff v2.2.1, which was run separately for each cell type. In order to control for genomic contamination, only those loci which contained at least one multi-exonic transcript or a single-exonic transcript with an FPKM > 1 in at least one subcellular fraction were considered for subsequent analyses (Additional file 5: Table S4).

### Linear correlations

We calculated the linear correlation between transcription (as scored by CAGE RPKM) or strength of accessible chromatin (as scored by the DHS RPKM) at chromatin state loci, non-chromatin state DHSs and bidirectionally transcribed-defined enhancers with the transcription (as scored by CAGE RPKM) from putative target, annotated GENCODE promoters across four cell types (Gm12878, HepG2, Huvec, K562). These regulatory loci were paired with target promoters as (a) the nearest promoter to each locus; (b) to all promoters within 500 kb of each locus; (c) to all promoters within the same physically interacting domain at each locus; (d) to all promoters which were determined to be physically interacting with each locus. Information on the location of each physically interacting domain and pairs of physically interacting loci were obtained from the GSE63525 series at the Gene Expression Omnibus [30]. If multiple target promoters were assigned to a given region, then the correlation which gave the lowest *p* value was considered. A positive correlation was recorded if the correlation coefficient was greater than 0 and *p* < 0.05. All other regions were considered to be nonsignificant. The uncertainty in the estimate of the percentage of positive correlations was determined by 1000 samplings of the data with replacement.

### Reporter assays

Enhancer and repressor element activity in K562 cells was estimated as the mean expression values obtained from a parallel reporter assay [26]. For this analysis, stably transcribed enhancers and repressed elements were identified as those with any evidence of stable transcription using CAGE originating from the entire chromatin state locus, regardless of DHS overlap. Bidirectional regions were defined as those with CAGE tags from both DNA strands, while unidirectional strands only had CAGE tags originating from one strand. Unstably transcribed elements were defined as those with no CAGE support over the locus but evidence of transcription from the GRO-cap dataset. The expression of sequence-scrambled controls for enhancer and repressor elements were not significantly different from each other (Mann-Whitney *p* = 0.20) and therefore these two categories were merged and considered as a single null expectation for the reporter activity measured from random DNA sequences.

Additional validations were performed in HepG2 cells (confirmed free of mycoplasma contamination with the Lonza MycoAlert kit). HepG2 cells were sourced from the Institute of Genetics and Molecular Medicine (Edinburgh) technical services. For our HepG2 reporter assays, DHSs from each chromatin state category which were more than 1 kb beyond RepeatMasker-marked regions and GENCODE gene annotations were randomly selected. PCR primers (Additional file 6: Table S5) with Kpn1 and EcoRV sites were used to amplify 500–1500 bp regions containing the DHS site from HepG2 genomic DNA. Amplicons were cloned into pGL4.26 vector post-restriction digest. For reporter assays, pGL4.26 constructs and the pRLTK plasmid were co-transfected into HepG2 cells with Lipofectamine-2000. Firefly and renilla luciferase activity was measured 48 hours post-transfection from three replicates using the Promega dual luciferase kit. The firefly luciferase signal was normalized by the renilla luciferase signal to reduce variability in transfection efficiency and an average reporter activity for the three replicates was then calculated relative to the empty pGL4.26 vector.

### Population genetics analysis

Derived allele frequencies were extracted from the deCODE whole-genome sequencing study of the Icelandic population [49]. Analogous to previous cross-species comparisons [7], for each category of transcript, all polymorphic sites overlapping exons and separately those overlapping introns from the same transcript class were aggregated. Additional file 5: Table S4 summarises the numbers of transcripts in each annotation category and whether they were single exon or multi-exon transcripts. Single nucleotide polymorphisms from both exonic and intronic classes were partitioned into rare (<1.5% population frequency) and common (>5% frequency) as done previously [32] and the odds ratio of rare:common between exonic and intronic sequence for a type to transcript (e.g., protein-coding, lincRNA, eRNA) was calculated. Odds ratio confidence intervals and *p* values were obtained using Fisher’s exact test (fisher.test function of R version 3.4.1).

## Additional files

Additional file 1: Figure S1. Bidirectional transcription (as measured by CAGE) initiation around DHSs in Gm12878 cells. **Figure S2.** Bidirectional transcription (as measured by CAGE) initiation around DHSs in K562 cells. **Figure S3.** Bidirectional transcription (as measured by CAGE) initiation around DHSs in HepG2 cells. **Figure S4.** Bidirectional transcription (as measured by CAGE) initiation around DHSs in Huvec cells. **Figure S5.** Bidirectional transcription initiation around DHSs in K562 cells. **Figure S6.** Bidirectional transcription (as measured by GRO-seq) initiation around DHSs in Gm12878 cells. **Figure S7.** Bidirectional transcription (as measured by GRO-seq) initiation around DHSs in K562 cells. **Figure S8.** Bidirectional transcription (as measured by PRO-seq) initiation around DHSs in K562 cells. **Figure S9.** Bidirectional transcription initiation (as measured by GRO-cap) across DHSs which do not overlap annotated promoters in two cell lines across various chromatin state annotations. **Figure S10.** Bidirectional transcription (as measured by CAGE) across DHSs which do not overlap annotated promoters in four cell lines across various chromatin state annotations. **Figure S11.** Productive transcription as measured by the elongation rates of transcripts initiating from various genome annotations and chromatin states. **Figure S12.** Correlations between chromatin state loci and bidirectionally transcribed-defined enhancers with the nearest annotated gene promoter. **Figure S13.** Correlations between chromatin state loci and bidirectionally transcribed-defined enhancers with annotated gene promoters within 500 kb. **Figure S14.** Correlations between chromatin state loci and bidirectionally transcribed-defined enhancers with annotated gene promoters within the same physical domain. **Figure S15.** Correlations between chromatin state loci and bidirectionally transcribed-defined enhancers with physically interacting annotated gene promoters. (PDF 1830 kb)

Additional file 2: Table S1. Counts of bidirectionally and unidirectionally transcribed DHSs defined as 250 bp around the midpoint across different chromatin state regions and gene annotations (mRNA/miRNA/lincRNA) as measured by GRO-cap, GRO-seq, and PRO-seq in K562 and, where available, Gm12878 cells. (DOC 88 kb)

Additional file 3: Table S2. Counts of bidirectionally and unidirectionally transcribed DHSs defined as 250 bp around the midpoint across different chromatin state regions and gene annotations (mRNA/miRNA/lincRNA) as measured by CAGE across cell types and subcellular fractions. (DOC 343 kb)

Additional file 4: Table S3. Number of chromatin state loci and bidirectionally transcribed-defined enhancers whose measure of stable transcription initiation and accessible chromatin is significantly correlated with transcription from putative annotated gene promoter targets as measured by either transcription initiation from the maximally expressed TSS (single TSS) or the sum of all annotated promoters (summed TSSs) associated with that gene. (DOC 51 kb)

Additional file 5: Table S4. Counts of single-exonic and multi-exonic transcripts built by Cufflinks and filtered as described in the “Methods” section (‘Transcriptome analysis’) for each annotation class analysed. (DOC 30 kb)

Additional file 6: Table S5. Primer sequences, coordinates, and reporter construct activities measured in HepG2 cells. (DOC 294 kb)

## Abbreviations

CAGE: Cap analysis of gene expression
DHS: DNase hypersensitivity site
eRNA: Enhancer RNA
FPKM: Fragments per kilobase exon per million reads mapped
GRO: Global run-on
lincRNA: Long intergenic noncoding RNA
RPKM: Reads per kilobase exon per million reads mapped
RPM: Reads per million reads mapped
TSS: Transcriptional start site
