# Supplementary material for: Bidirectional transcription initiation marks accessible chromatin and is not specific to enhancers
Source: Genome Biol. 2017 Dec 28;18:242. doi: 10.1186/s13059-017-1379-8 (PMC5747114; doi:10.1186/s13059-017-1379-8)
Supplement: Supplementary file 1 — Bidirectional transcription (as measured by CAGE) initiation around DHSs in Gm12878 cells. Figure S2. Bidirectional transcription (as measured by CAGE) initiation around DHSs in K562 cells. Figure S3. Bidirectional transcription (as measured by CAGE) initiation around DHSs in HepG2 cells. Figure S4. Bidirectional transcription (as measured by CAGE) initiation around DHSs in Huvec cells. Figure S5. Bidirectional transcription initiation around DHSs in K562 cells. Figure S6. Bidirectional transcription (as measured by GRO-seq) initiation around DHSs in Gm12878 cells. Figure S7. Bidirectional transcription (as measured by GRO-seq) initiation around DHSs in K562 cells. Figure S8. Bidirectional transcription (as measured by PRO-seq) initiation around DHSs in K562 cells. Figure S9. Bidirectional transcription initiation (as measured by GRO-cap) across DHSs which do not overlap annotated promoters in two cell lines across various chromatin state annotations. Figure S10. Bidirectional transcription (as measured by CAGE) across DHSs which do not overlap annotated promoters in four cell lines across various chromatin state annotations. Figure S11. Productive transcription as measured by the elongation rates of transcripts initiating from various genome annotations and chromatin states. Figure S12. Correlations between chromatin state loci and bidirectionally transcribed-defined enhancers with the nearest annotated gene promoter. Figure S13. Correlations between chromatin state loci and bidirectionally transcribed-defined enhancers with annotated gene promoters within 500 kb. Figure S14. Correlations between chromatin state loci and bidirectionally transcribed-defined enhancers with annotated gene promoters within the same physical domain. Figure S15. Correlations between chromatin state loci and bidirectionally transcribed-defined enhancers with physically interacting annotated gene promoters. (PDF 1830 kb) [file 13059_2017_1379_MOESM1_ESM.pdf]

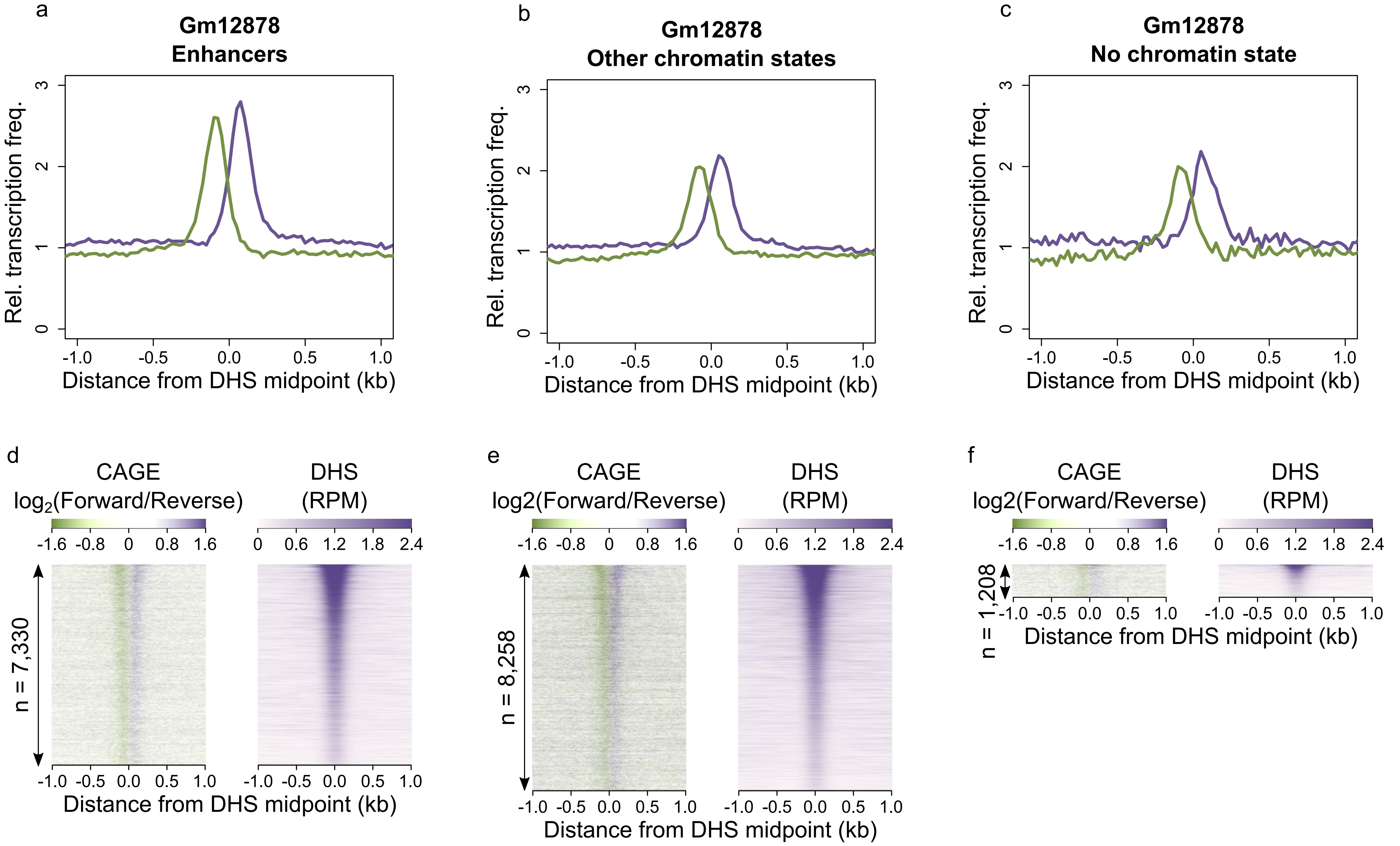

Figure S1 : Bidirectional transcription (as measured by **CAGE**) initiates around DHSs but is not a specific mark of active enhancers in **Gm12878** cells. (a-c) The fold-change in transcription frequency (fraction of loci with evidence of transcription initiation) for sites with transcription initiation signal in 25 bp consecutive windows around DHS midpoints (x = 0) relative to the mean transcription frequency in the flanking regions: 500 to 1,000 bp from the DHS midpoint. In all panels, only DHSs that do not overlap annotated promoters were included. Purple lines consider transcription initiation from the forward strand and green lines show transcription initiation from the reverse strand. (d-f) Heatmaps of **CAGE** signal as measured by log2(Forward/Reverse) RPM and DNase hypersensitivity as measured by RPM around DHS midpoints for the same chromatin state annotations described in a-c. Rows are ranked by the DNase hypersensitivity signal (RPM). The height of each heatmap corresponds to the total number of DHSs with evidence for outward-facing bidirectional transcription initiation within 250 bp of the DHS midpoint, i.e. at least one **CAGE** tag on the reverse strand within 250 bp downstream of the midpoint and at least one tag on the forward strand within 250 bp upstream of the midpoint, so that shading density is directly comparable between plots.

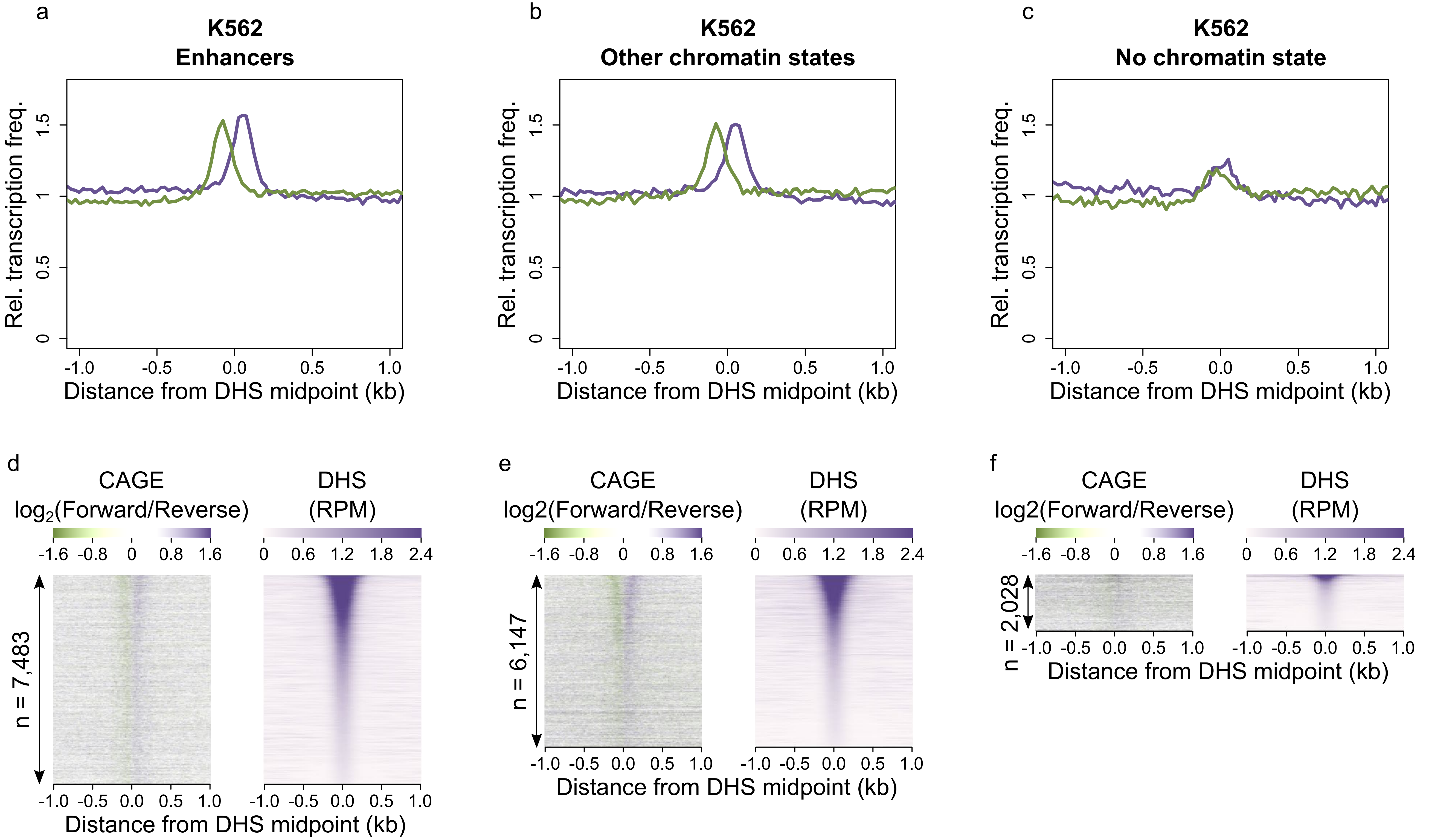

Figure S2 : Bidirectional transcription (as measured by **CAGE**) initiates around DHSs but is not a specific mark of active enhancers in **K562** cells. (a-c) The fold-change in transcription frequency (fraction of loci with evidence of transcription initiation) for sites with transcription initiation signal in 25 bp consecutive windows around DHS midpoints (x = 0) relative to the mean transcription frequency in the flanking regions: 500 to 1,000 bp from the DHS midpoint. In all panels, only DHSs that do not overlap annotated promoters were included. Purple lines consider transcription initiation from the forward strand and green lines show transcription initiation from the reverse strand. (d-f) Heatmaps of **CAGE** signal as measured by log2(Forward/ Reverse) RPM and DNase hypersensitivity as measured by RPM around DHS midpoints for the same chromatin state annotations described in a-c. Rows are ranked by the DNase hypersensitivity signal (RPM). The height of each heatmap corresponds to the total number of DHSs with evidence for outward-facing bidirectional transcription initiation within 250 bp of the DHS midpoint, i.e. at least one **CAGE** tag on the reverse strand within 250 bp downstream of the midpoint and at least one tag on the forward strand within 250 bp upstream of the midpoint, so that shading density is directly comparable between plots.

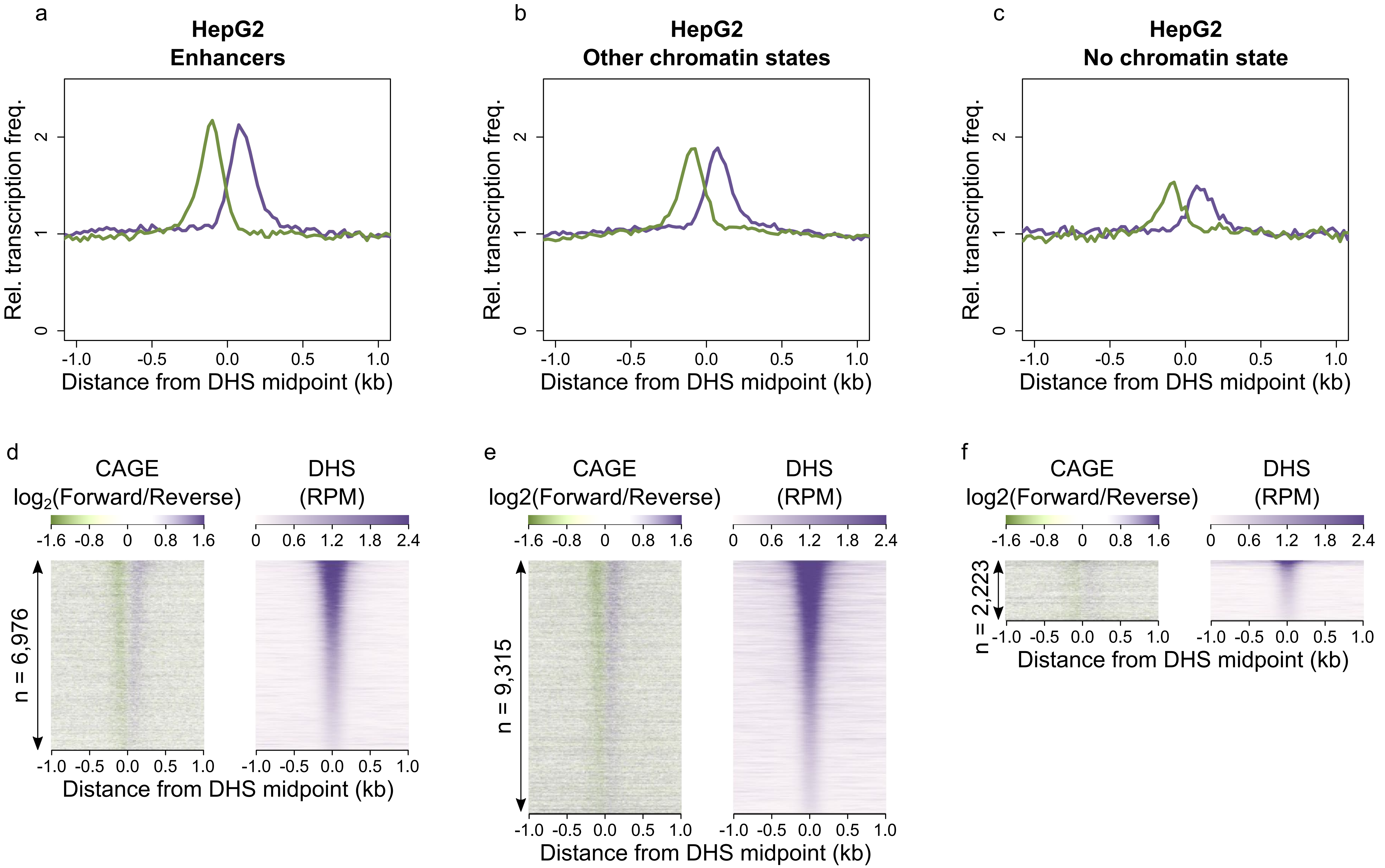

Figure S3: Bidirectional transcription (as measured by **CAGE**) initiates around DHSs but is not a specific mark of active enhancers in **HepG2** cells. (a-c) The fold-change in transcription frequency (fraction of loci with evidence of transcription initiation) for sites with transcription initiation signal in 25 bp consecutive windows around DHS midpoints (x = 0) relative to the mean transcription frequency in the flanking regions: 500 to 1,000 bp from the DHS midpoint. In all panels, only DHSs that do not overlap annotated promoters were included. Purple lines consider transcription initiation from the forward strand and green lines show transcription initiation from the reverse strand. (d-f) Heatmaps of **CAGE** signal as measured by log2(Forward/Reverse) RPM and DNase hypersensitivity as measured by RPM around DHS midpoints for the same chromatin state annotations described in a-c. Rows are ranked by the DNase hypersensitivity signal (RPM). The height of each heatmap corresponds to the total number of DHSs with evidence for outward-facing bidirectional transcription initiation within 250 bp of the DHS midpoint, i.e. at least one **CAGE** tag on the reverse strand within 250 bp downstream of the midpoint and at least one tag on the forward strand within 250 bp upstream of the midpoint, so that shading density is directly comparable between plots.

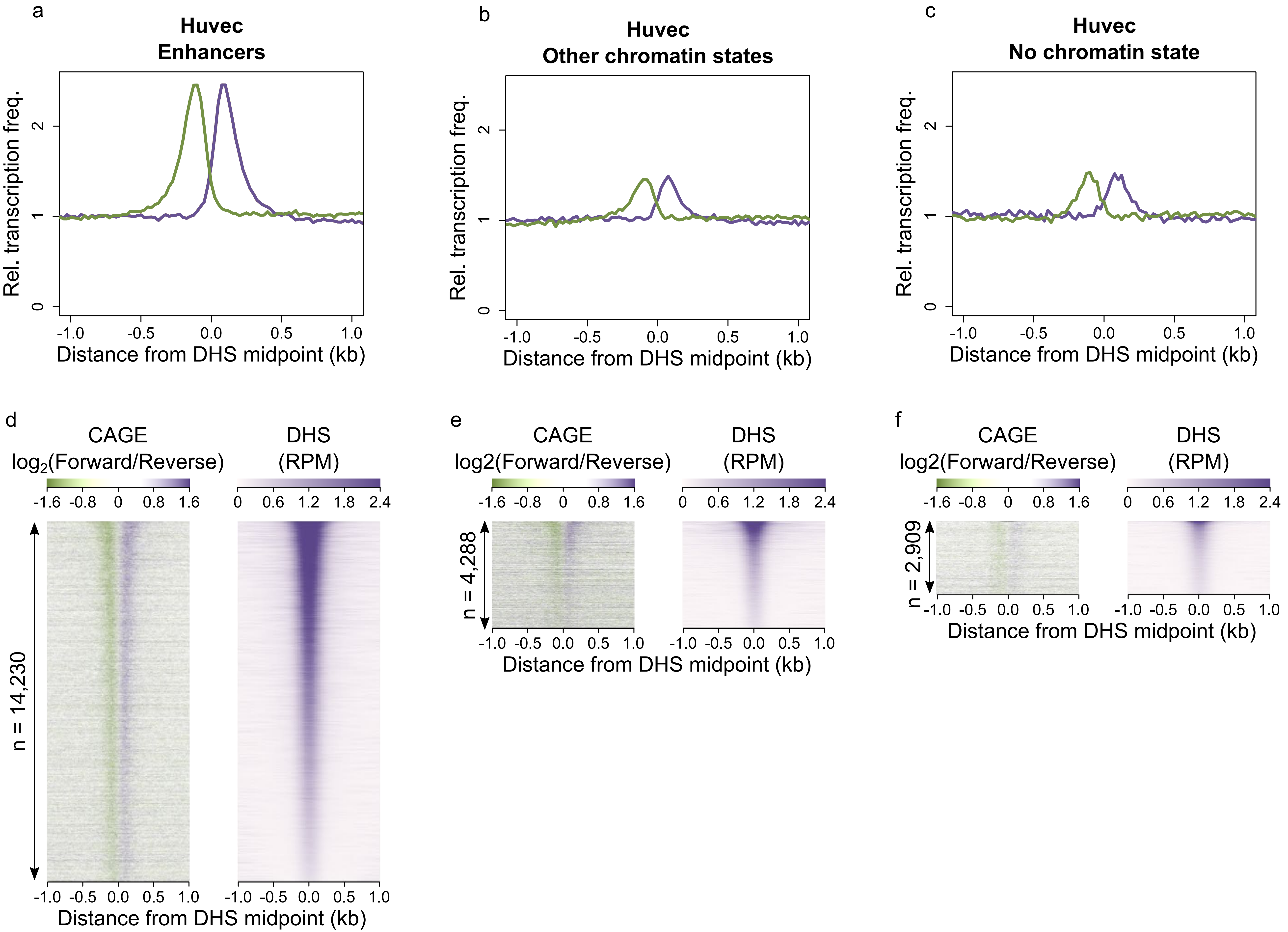

Figure S4: Bidirectional transcription (as measured by **CAGE**) initiates around DHSs but is not a specific mark of active enhancers in **Huvec** cells. (a-c) The fold-change in transcription frequency (fraction of loci with evidence of transcription initiation) for sites with transcription initiation signal in 25 bp consecutive windows around DHS midpoints (x = 0) relative to the mean transcription frequency in the flanking regions: 500 to 1,000 bp from the DHS midpoint. In all panels, only DHSs that do not overlap annotated promoters were included. Purple lines consider transcription initiation from the forward strand and green lines show transcription initiation from the reverse strand. (d-f) Heatmaps of **CAGE** signal as measured by log2(Forward/Reverse) RPM and DNase hypersensitivity as measured by RPM around DHS midpoints for the same chromatin state annotations described in a-c. Rows are ranked by the DNase hypersensitivity signal (RPM). The height of each heatmap corresponds to the total number of DHSs with evidence for outward-facing bidirectional transcription initiation within 250 bp of the DHS midpoint, i.e. at least one **CAGE** tag on the reverse strand within 250 bp downstream of the midpoint and at least one tag on the forward strand within 250 bp upstream of the midpoint, so that shading density is directly comparable between plots.

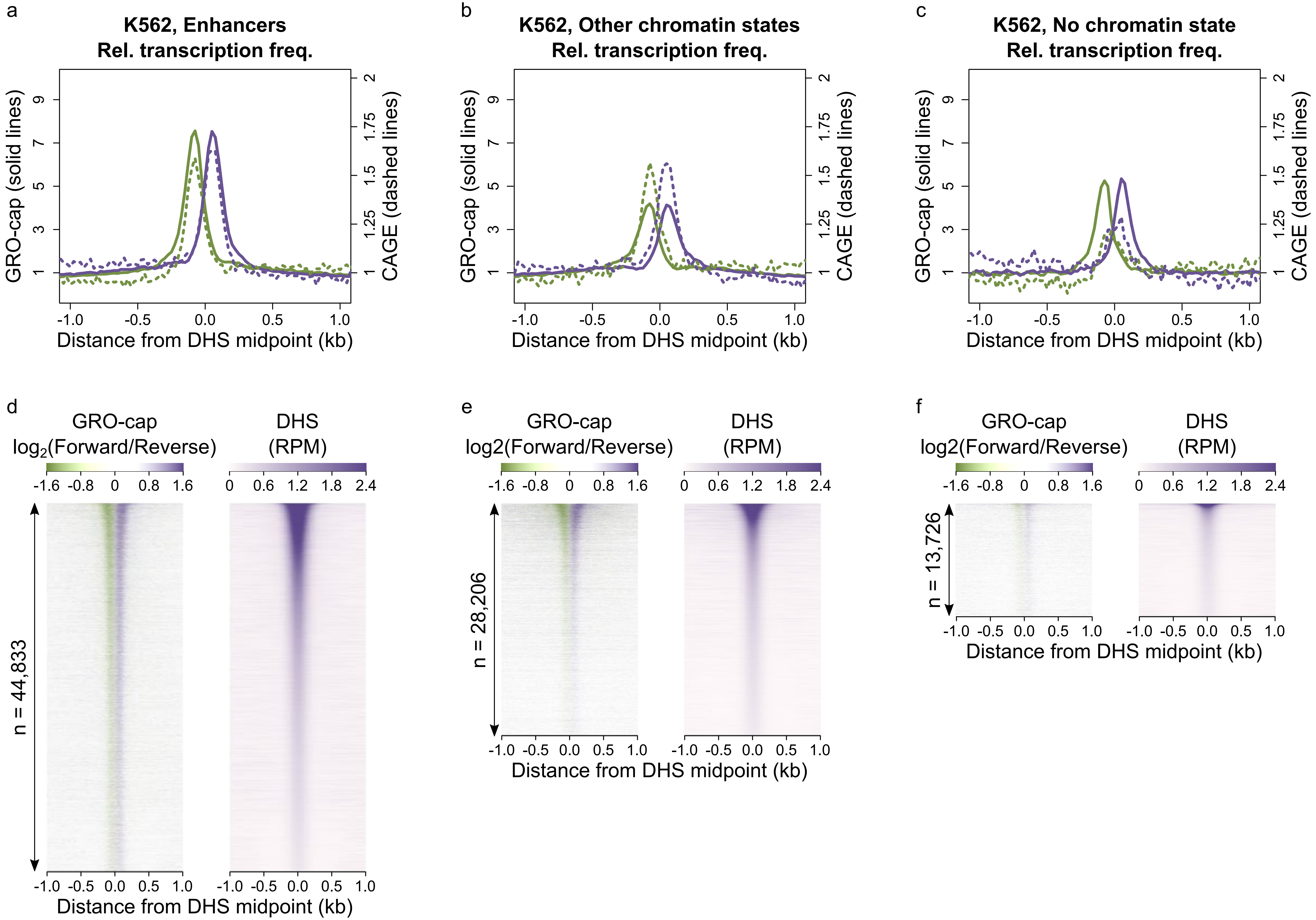

Figure S5: Bidirectional transcription initiates around DHSs but is not a specific mark of active enhancers in **K562** cells. (a-c) The fold-change in transcription frequency (fraction of loci with evidence of transcription initiation) for sites with transcription initiation signal in 25 bp consecutive windows around DHS midpoints (x = 0) relative to the mean transcription frequency in the flanking regions: 500 to 1,000 bp from the DHS midpoint. Total bidirectional transcription initiation across DHSs in **K562** cells as measured by **GRO-cap** is shown by the solid lines while stable bidirectional transcription initiation as measured by **CAGE** is shown by the dashed lines. In all panels, only DHSs that do not overlap annotated promoters were included. Purple lines consider transcription initiation from the forward strand and green lines show transcription initiation from the reverse strand. (d-f) Heatmaps of **GRO-cap** signal as measured by log2(Forward/Reverse) RPM and DNase hypersensitivity as measured by RPM around DHS midpoints for the same chromatin state annotations described in a-c. Rows are ranked by the DNase hypersensitivity signal (RPM). The height of each heatmap corresponds to the total number of DHSs which generated the plot as shown on the y-axis so that shading density is directly comparable between plots.

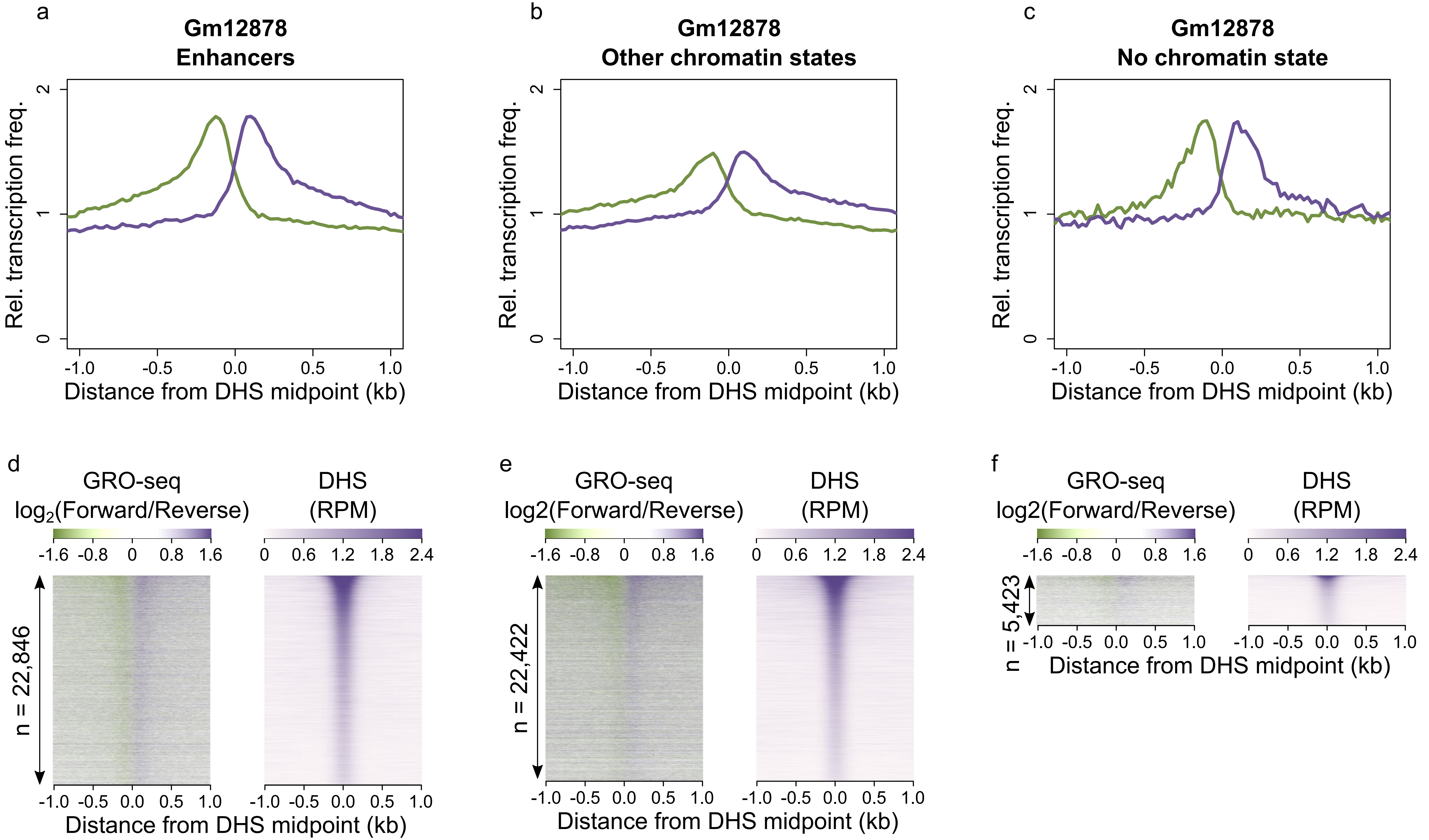

Figure S6: Bidirectional transcription (as measured by **GRO-seq**) initiates around DHSs but is not a specific mark of active enhancers in **Gm12878** cells. (a-c) The fold-change in transcription frequency (fraction of loci with evidence of transcription initiation) for sites with transcription initiation signal in 25 bp consecutive windows around DHS midpoints (x = 0) relative to the mean transcription frequency in the flanking regions: 500 to 1,000 bp from the DHS midpoint. In all panels, only DHSs that do not overlap annotated promoters were included. Purple lines consider transcription initiation from the forward strand and green lines show transcription initiation from the reverse strand. (d-f) Heatmaps of **GRO-seq** signal as measured by log2(Forward/ Reverse) RPM and DNase hypersensitivity as measured by RPM around DHS midpoints for the same chromatin state annotations described in a-c. Rows are ranked by the DNase hypersensitivity signal (RPM). The height of each heatmap corresponds to the total number of DHSs which generated the plot as shown on the y-axis so that shading density is directly comparable between plots.

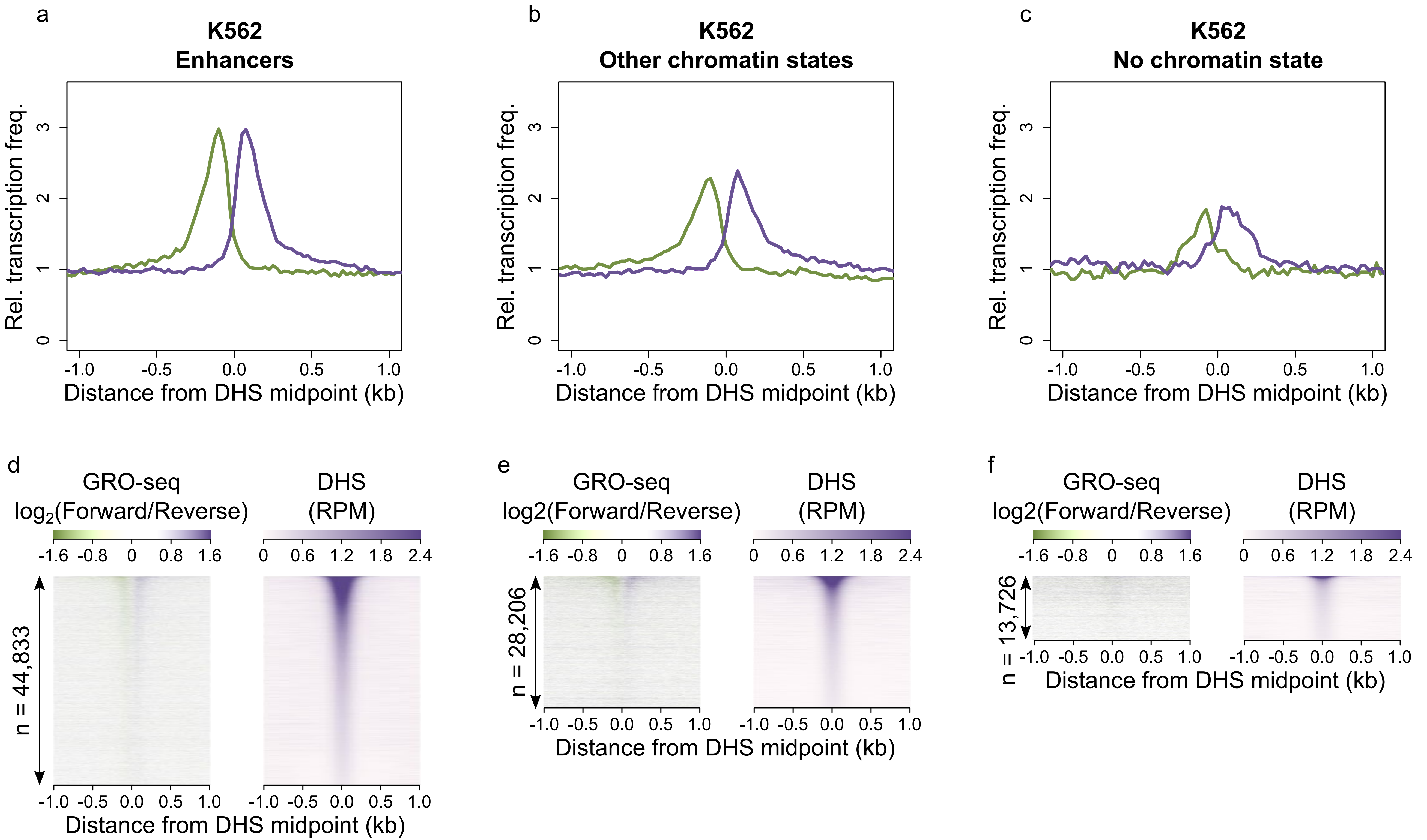

Figure S7: Bidirectional transcription (as measured by **GRO-seq**) initiates around DHSs but is not a specific mark of active enhancers in **K562** cells. (a-c) The fold-change in transcription frequency (fraction of loci with evidence of transcription initiation) for sites with transcription initiation signal in 25 bp consecutive windows around DHS midpoints (x = 0) relative to the mean transcription frequency in the flanking regions: 500 to 1,000 bp from the DHS midpoint. In all panels, only DHSs that do not overlap annotated promoters were included. Purple lines consider transcription initiation from the forward strand and green lines show transcription initiation from the reverse strand. (d-f) Heatmaps of **GRO-seq** signal as measured by log2(Forward/Reverse) RPM and DNase hypersensitivity as measured by RPM around DHS midpoints for the same chromatin state annotations described in a-c. Rows are ranked by the DNase hypersensitivity signal (RPM). The height of each heatmap corresponds to the total number of DHSs which generated the plot as shown on the y-axis so that shading density is directly comparable between plots.

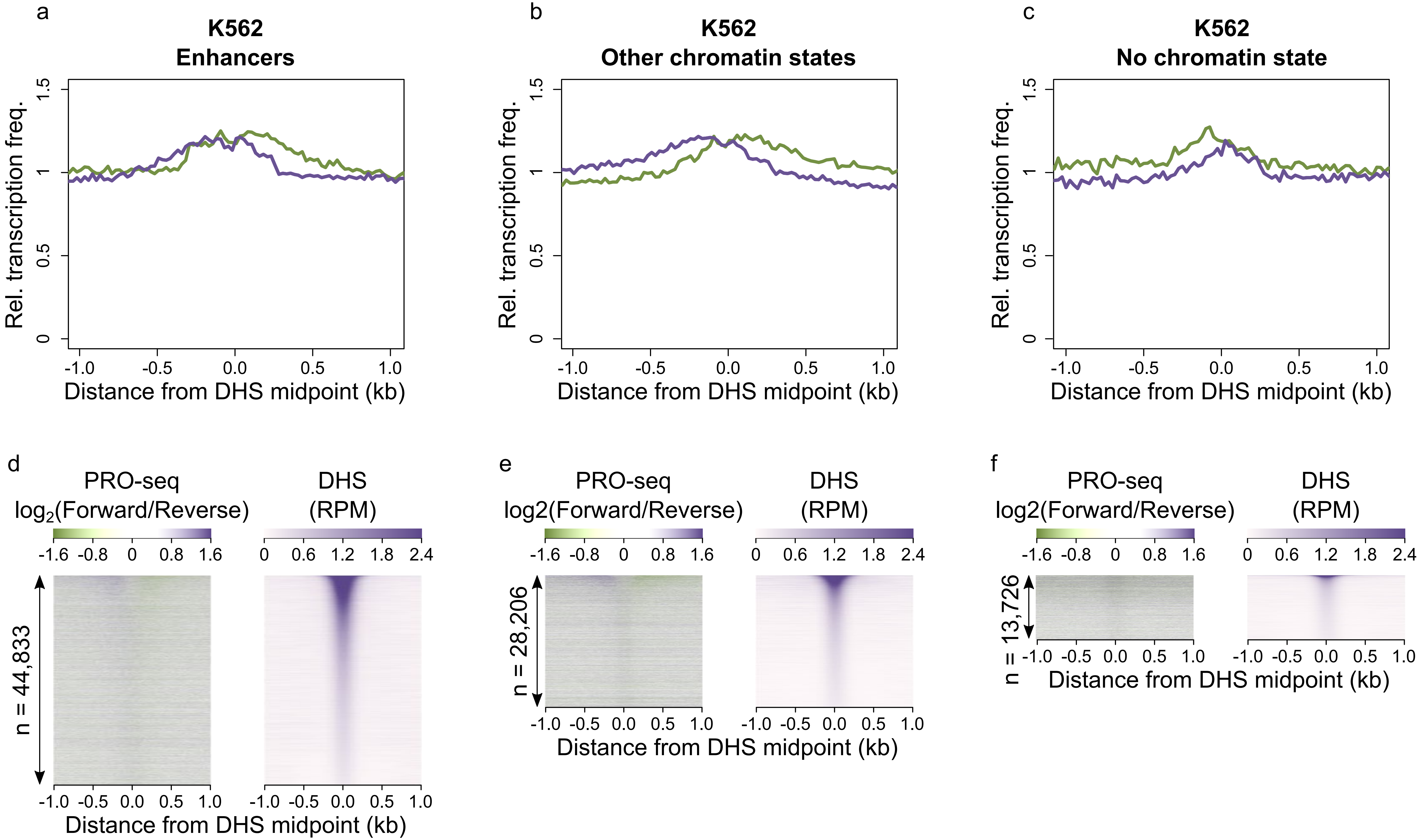

Figure S8: Bidirectional transcription (as measured by **PRO-seq**) initiates around DHSs but is not a specific mark of active enhancers in **K562** cells. (a-c) The fold-change in transcription frequency (fraction of loci with evidence of transcription initiation) for sites with transcription initiation signal in 25 bp consecutive windows around DHS midpoints (x = 0) relative to the mean transcription frequency in the flanking regions: 500 to 1,000 bp from the DHS midpoint. In all panels, only DHSs that do not overlap annotated promoters were included. Purple lines consider transcription initiation from the forward strand and green lines show transcription initiation from the reverse strand. (d-f) Heatmaps of **PRO-seq** signal as measured by log2(Forward/ Reverse) RPM and DNase hypersensitivity as measured by RPM around DHS midpoints for the same chromatin state annotations described in a-c. Rows are ranked by the DNase hypersensitivity signal (RPM). The height of each heatmap corresponds to the total number of DHSs which generated the plot as shown on the y-axis so that shading density is directly comparable between plots.

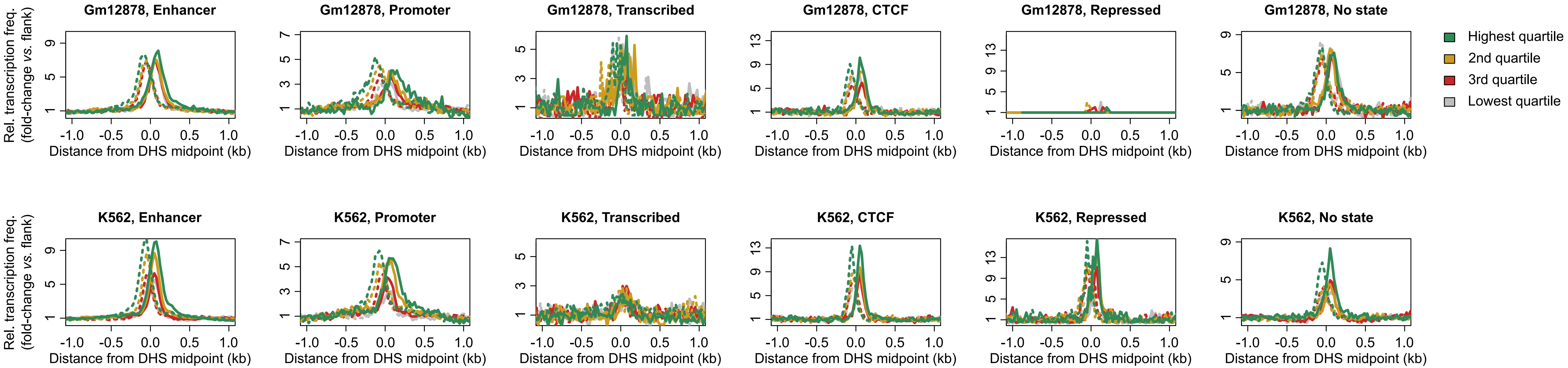

Figure S9: Bidirectional transcription initiation (as measured by **GRO-cap**) across DHSs which do not overlap annotated promoters in two cell lines across various chromatin state annotations. Y-axes show the fold-change in transcription frequency (fraction of loci with evidence of transcription initiation) for sites with transcription initiation signal in 25 bp windows around DHS midpoints (x = 0) relative to the mean transcription in the flanking regions: 500 to 1,000 bp from the DHS midpoint. DHSs for each cell line were split into quartiles of increasing DHS peak height. The solid lines consider **GRO-cap** tags from the positive strand only and the dashed lines consider **GRO-cap** tags from the negative strand only.

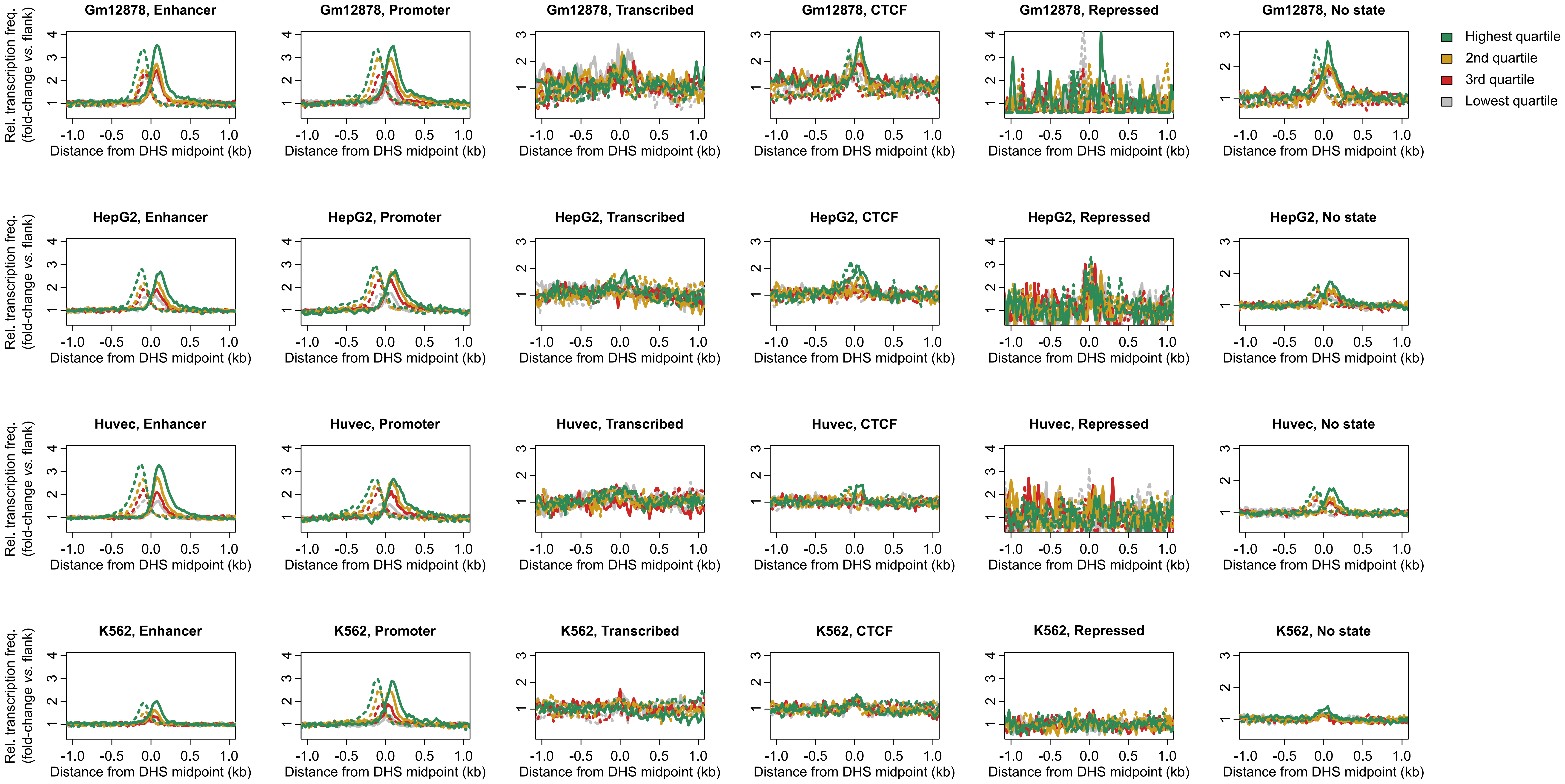

Figure S10: Bidirectional transcription (as measured by **CAGE**) across DHSs which do not overlap annotated promoters in four cell lines across various chromatin state annotations. Y-axes show the fold-change in transcription frequency (fraction of loci with evidence of transcription initiation) for sites with transcription initiation signal in 25 bp windows around DHS midpoints (x = 0) relative to the mean transcription in the flanking regions: 500 to 1,000 bp from the DHS midpoint. DHSs for each cell line were split into quartiles of increasing DHS peak height. The solid lines consider **CAGE** tags from the positive strand only and the dashed lines consider **CAGE** tags from the negative strand only.

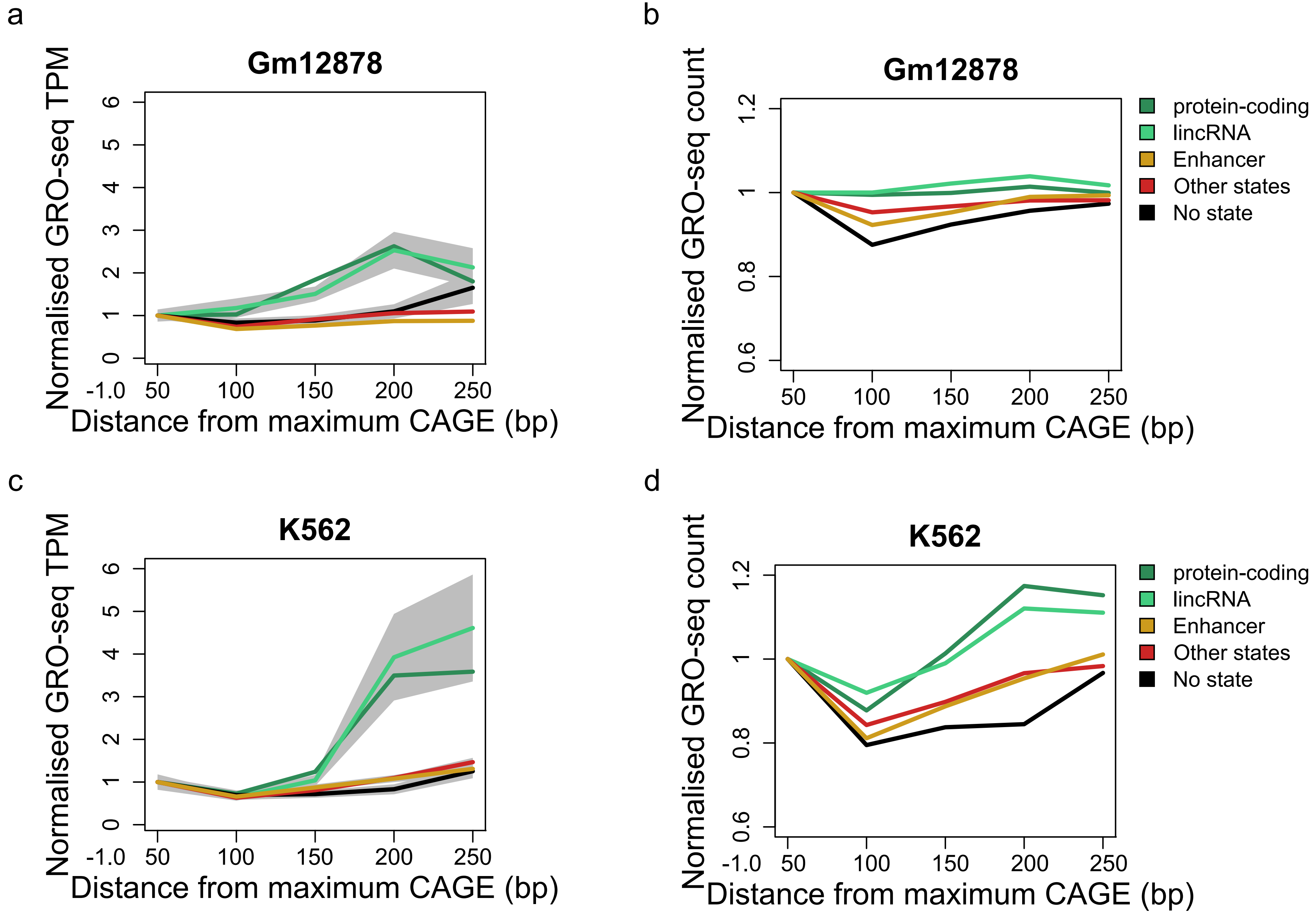

Figure S11: Productive transcription as measured by the elongation rates of transcripts initiating from various genome annotations (protein-coding and lincRNA TSSs) and chromatin states (enhancers, all other states, and outside chromatin state maps). The GRO-seq coverage was measured in 50 bp windows extending from the site with maximum CAGE signal within each annotation and then normalised to the signal in the first 50 bp window for each annotation class. (a, c) The mean strength of GRO-seq signal as measured by TPM, with the grey boundaries corresponding to the standard error of the mean for each window. (b, d) The normalised fraction of windows which contained at least one GRO-seq read for each annotation class.

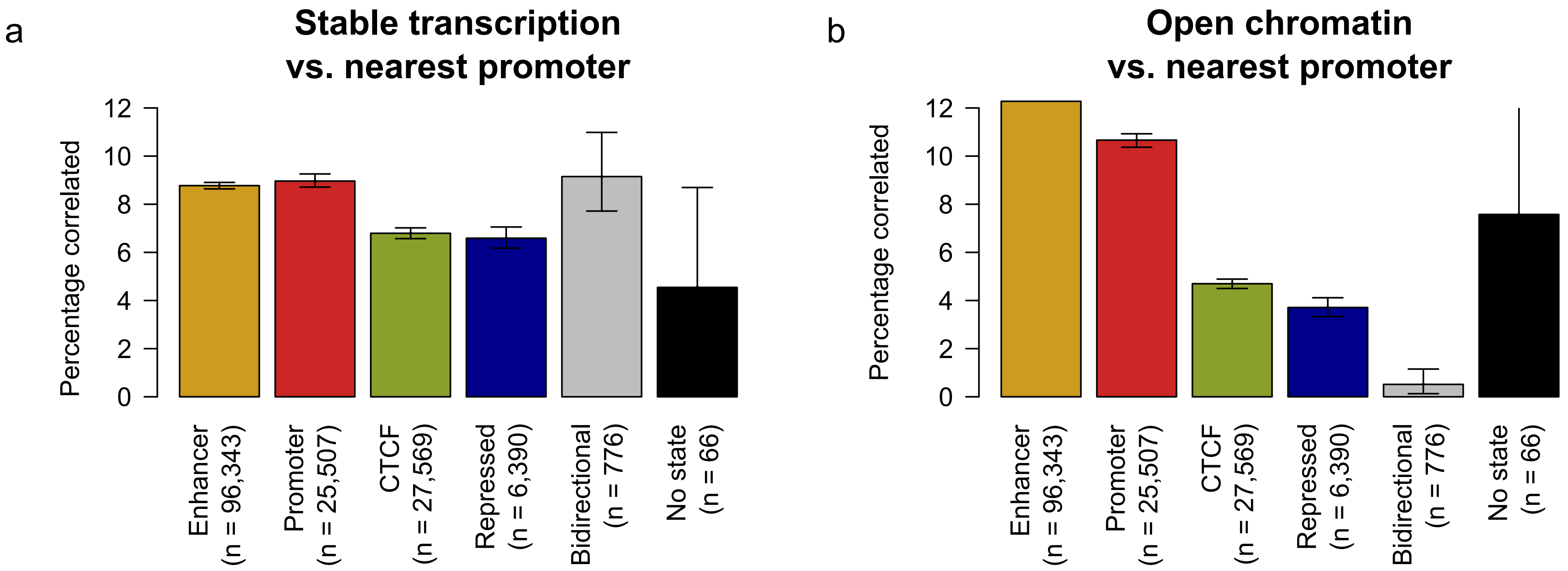

Figure S12: (a) The percentage of chromatin state loci and bidirectionally transcribed-defined enhancers whose measure of stable transcription initiation is significantly correlated with the summed transcription initiation across the nearest annotated gene promoter. The error bars represent the 95% confidence interval from 1,000 samplings of the data with replacement, while the numbers below each bar denote the number of loci tested for a significant correlation. (b) As for a, but the correlations being considered are between the level of DHS signal of chromatin state loci and bidirectionally transcribed-defined enhancers and the summed transcription initiation from the nearest annotated gene promoter.

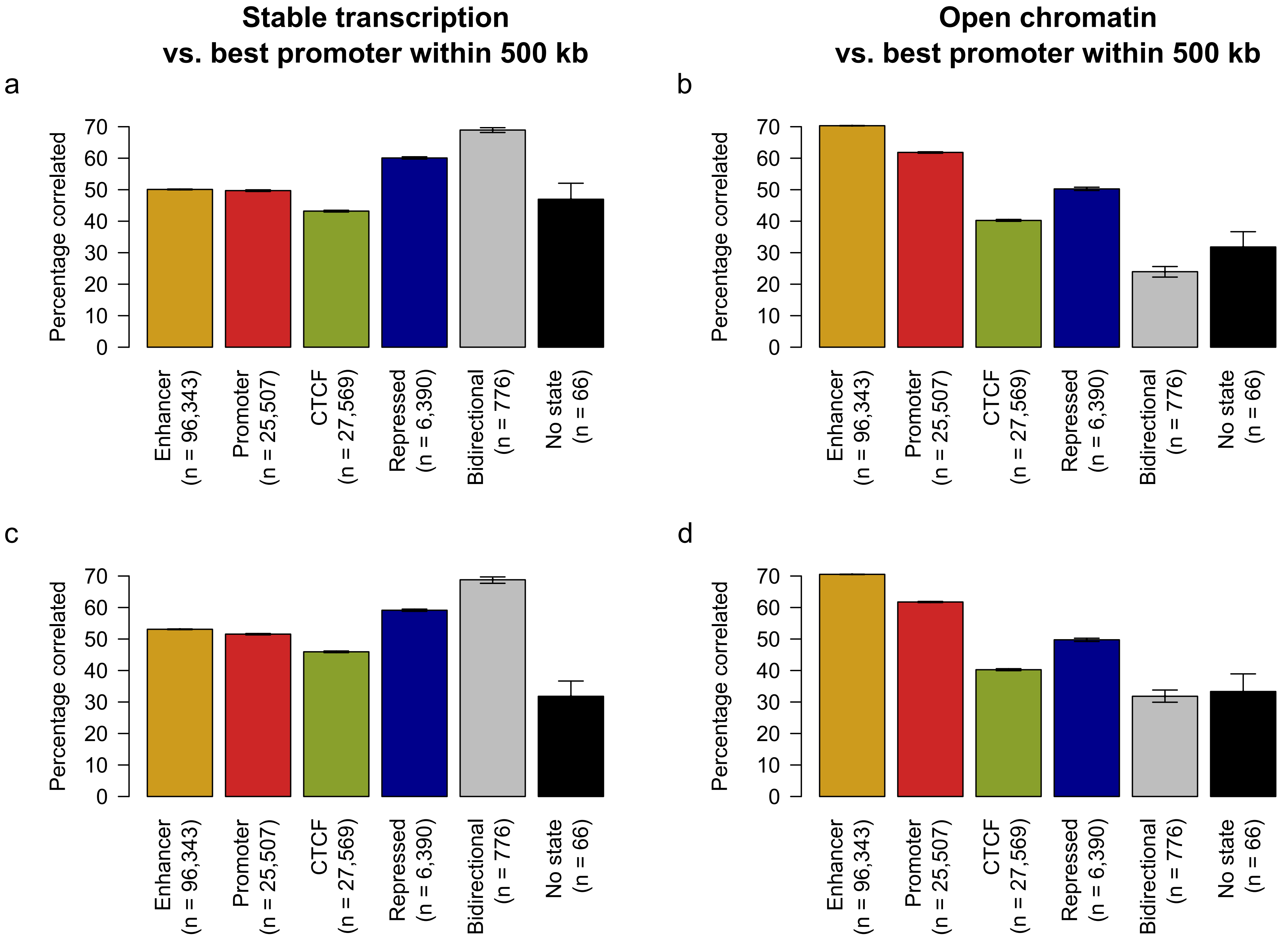

Figure S13: (a) The percentage of chromatin state loci and bidirectionally transcribed-defined enhancers whose measure of stable transcription initiation is significantly correlated with transcription initiation from at least one annotated gene promoter within 500 kb. The error bars represent the 95% confidence interval from 1,000 samplings of the data with replacement, while the numbers below each bar denote the number of loci tested for a significant correlation. (b) As for a, but the correlations being considered are between the level of DHS signal of chromatin state loci and bidirectionally transcribed-defined enhancers and transcription initiation from annotated gene promoters within 500 kb. (c) The percentage of chromatin state loci and bidirectionally transcribed-defined enhancers whose measure of stable transcription initiation is significantly correlated with the summed transcription initiation across at least one annotated gene promoter within 500 kb. The error bars represent the 95% confidence interval from 1,000 samplings of the data with replacement, while the numbers below each bar denote the number of loci tested for a significant correlation. (d) As for c, but the correlations being considered are between the level of DHS signal of chromatin state loci and bidirectionally transcribed-defined enhancers and the summed transcription initiation from annotated gene promoters within 500 kb.

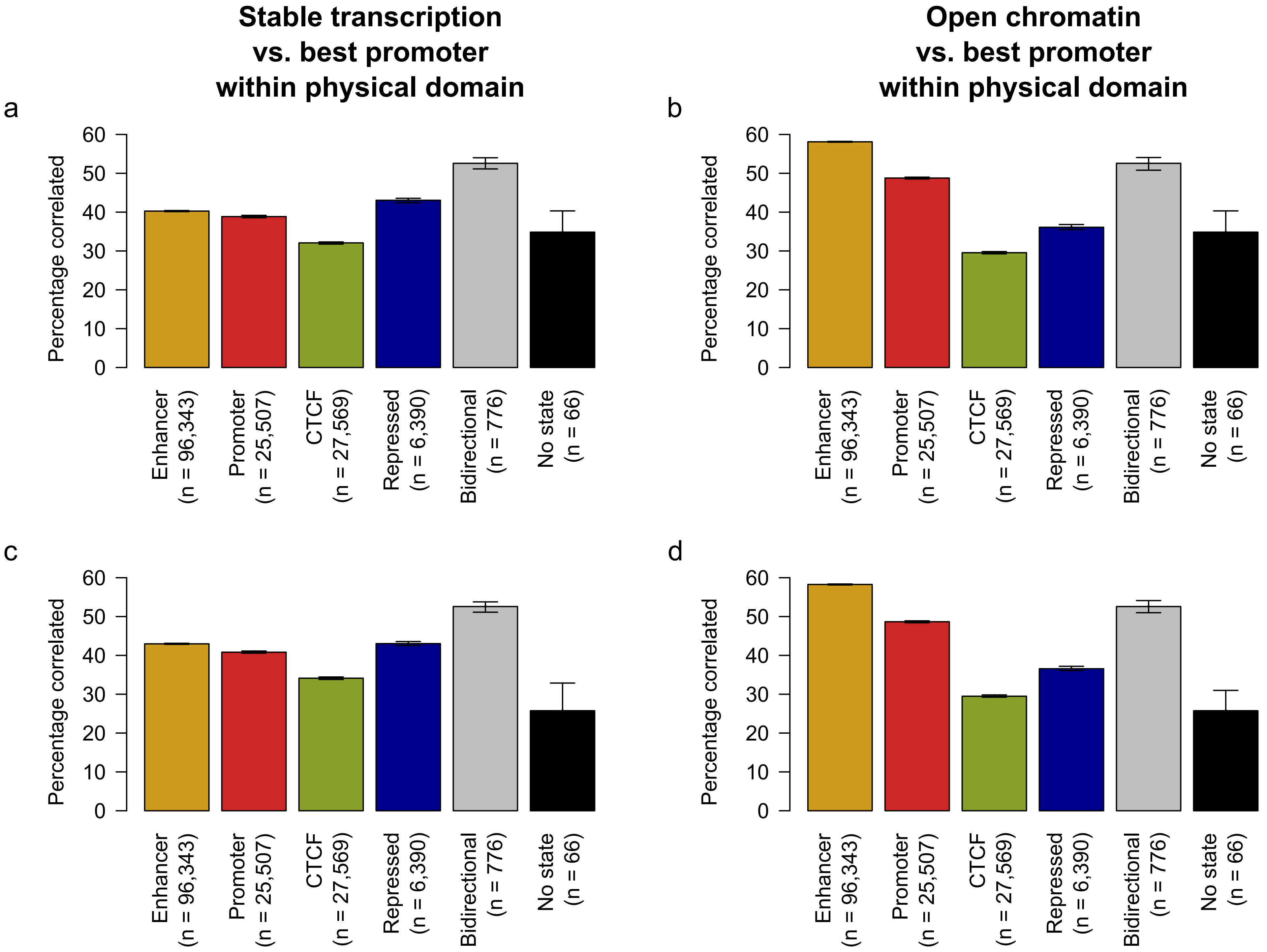

Figure S14: (a) The percentage of chromatin state loci and bidirectionally transcribed-defined enhancers whose measure of stable transcription initiation is significantly correlated with transcription initiation from at least one annotated gene promoter within the same physical domain as defined by Rao et al. 2014. The error bars represent the 95% confidence interval from 1,000 samplings of the data with replacement, while the numbers below each bar denote the number of loci tested for a significant correlation. (b) As for a, but the correlations being considered are between the level of DHS signal of chromatin state loci and bidirectionally transcribed-defined enhancers and transcription initiation from annotated gene promoters within the same domain. (c) The percentage of chromatin state loci and bidirectionally transcribed-defined enhancers whose measure of stable transcription initiation is significantly correlated with the summed transcription initiation across at least one annotated gene promoter within the same domain. The error bars represent the 95% confidence interval from 1,000 samplings of the data with replacement, while the numbers below each bar denote the number of loci tested for a significant correlation. (d) As for c, but the correlations being considered are between the level of DHS signal of chromatin state loci and bidirectionally transcribed-defined enhancers and the summed transcription initiation from annotated gene promoters within the same domain.

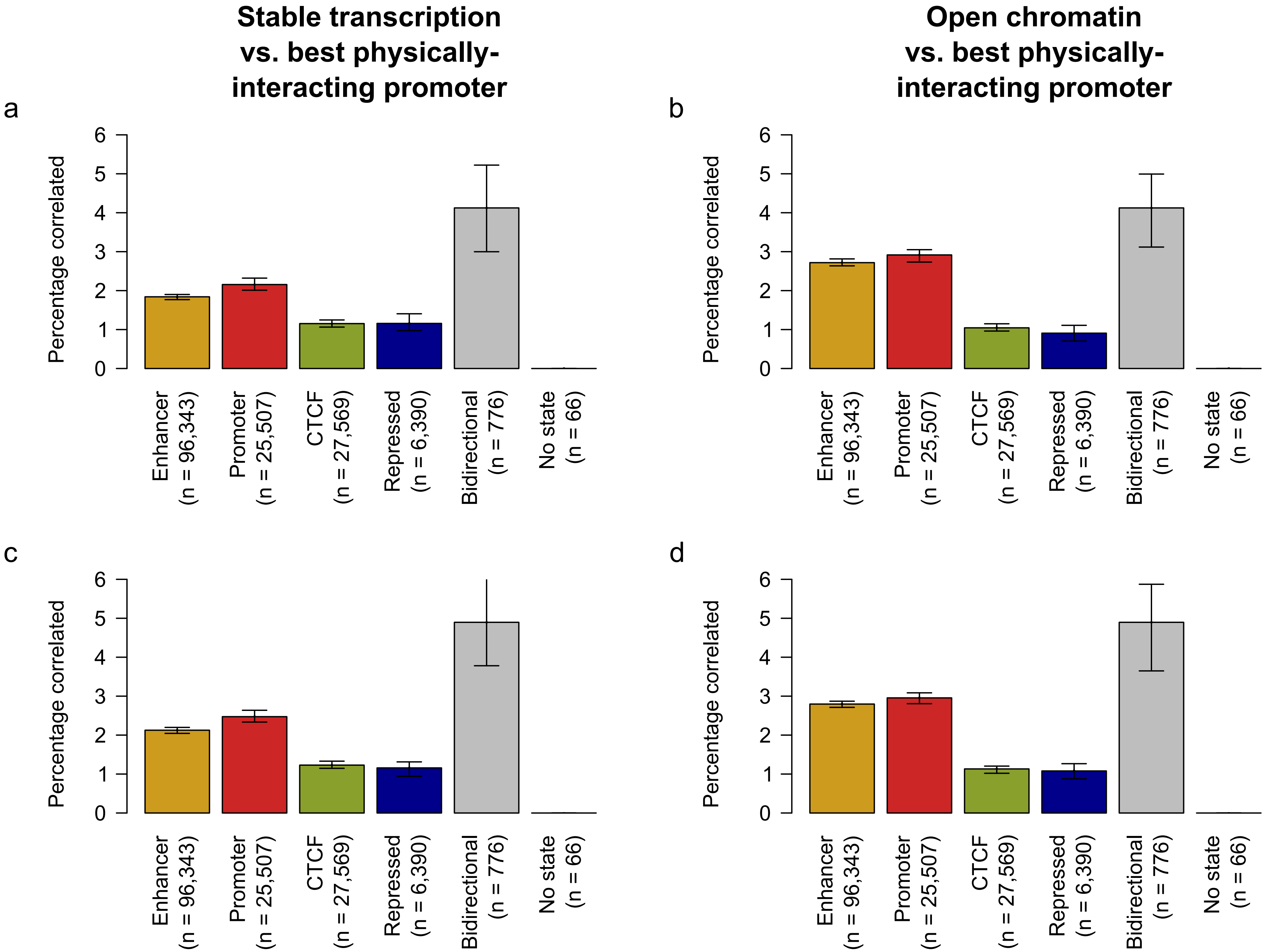

Figure S15: (a) The percentage of chromatin state loci and bidirectionally transcribed-defined enhancers whose measure of stable transcription initiation is significantly correlated with transcription initiation from at least one physically-interacting, annotated gene promoter as identified by Rao et al. 2014. The error bars represent the 95% confidence interval from 1,000 samplings of the data with replacement, while the numbers below each bar denote the number of loci tested for a significant correlation. (b) As for a, but the correlations being considered are between the level of DHS signal of chromatin state loci and bidirectionally transcribed-defined enhancers and transcription initiation from physically-interacting, annotated gene promoters. (c) The percentage of chromatin state loci and bidirectionally transcribed-defined enhancers whose measure of stable transcription initiation is significantly correlated with the summed transcription initiation across at least one physically-interacting, annotated gene promoter. The error bars represent the 95% confidence interval from 1,000 samplings of the data with replacement, while the numbers below each bar denote the number of loci tested for a significant correlation. (d) As for c, but the correlations being considered are between the level of DHS signal of chromatin state loci and bidirectionally transcribed-defined enhancers and the summed transcription initiation from physically-interacting, annotated gene promoters.
