# Supplementary material for: Bidirectional transcription initiation marks accessible chromatin and is not specific to enhancers
Source: Genome Biol. 2017 Dec 28;18:242. doi: 10.1186/s13059-017-1379-8 (PMC5747114; doi:10.1186/s13059-017-1379-8)
Supplement: Supplementary file 2 — Counts of bidirectionally and unidirectionally transcribed DHSs defined as 250 bp around the midpoint across different chromatin state regions and gene annotations (mRNA/miRNA/lincRNA) as measured by GRO-cap, GRO-seq, and PRO-seq in K562 and, where available, Gm12878 cells. (DOC 88 kb) [file 13059_2017_1379_MOESM2_ESM.doc]

**Table S1**: Counts of bidirectionally and unidirectionally transcribed DHSs defined as ± 250 bp around the midpoint across different chromatin state regions and gene annotations (mRNA/miRNA/lincRNA) as measured by GRO-cap, GRO-seq and PRO-seq in K562 and, where available, Gm12878 cells.

| Cell | Sample | Feature | Total | Bidirectional | Percent | Unidirectional | Percent |
| --- | --- | --- | --- | --- | --- | --- | --- |
| Gm12878 | GRO-cap | Enhancer | 22,846 | 8,272 | (36.21%) | 8,295 | (36.31%) |
| Gm12878 | GRO-cap | Promoter | 10,566 | 6,995 | (66.20%) | 2,693 | (25.49%) |
| Gm12878 | GRO-cap | Transcribed | 1,106 | 125 | (11.3%) | 431 | (38.97%) |
| Gm12878 | GRO-cap | CTCF | 10,779 | 572 | (5.31%) | 2,558 | (23.73%) |
| Gm12878 | GRO-cap | Repressed | 113 | 1 | (0.88%) | 27 | (23.89%) |
| Gm12878 | GRO-cap | Bidirectional | 7,064 | 3,989 | (56.47%) | 1,929 | (27.31%) |
| Gm12878 | GRO-cap | No state | 5,423 | 735 | (13.55%) | 1,534 | (28.29%) |
| Gm12878 | GRO-cap | mRNA | 25,074 | 23,924 | (95.41%) | 788 | (3.14%) |
| Gm12878 | GRO-cap | miRNA | 135 | 111 | (82.22%) | 12 | (8.89%) |
| Gm12878 | GRO-cap | lincRNA | 641 | 550 | (85.80%) | 31 | (4.84%) |
| Gm12878 | GRO-seq | Enhancer | 22,846 | 11,346 | (49.66%) | 8,275 | (36.22%) |
| Gm12878 | GRO-seq | Promoter | 10,566 | 8,452 | (79.99%) | 1,931 | (18.28%) |
| Gm12878 | GRO-seq | Transcribed | 1,106 | 339 | (30.65%) | 744 | (67.27%) |
| Gm12878 | GRO-seq | CTCF | 10,779 | 1,532 | (14.21%) | 4,870 | (45.18%) |
| Gm12878 | GRO-seq | Repressed | 113 | 7 | (6.19%) | 29 | (25.66%) |
| Gm12878 | GRO-seq | Bidirectional | 7,064 | 4,623 | (65.44%) | 1,744 | (24.69%) |
| Gm12878 | GRO-seq | No state | 5,423 | 1,322 | (24.38%) | 2,213 | (40.81%) |
| Gm12878 | GRO-seq | mRNA | 25,074 | 24,281 | (96.84%) | 509 | (2.03%) |
| Gm12878 | GRO-seq | miRNA | 135 | 124 | (91.85%) | 8 | (5.93%) |
| Gm12878 | GRO-seq | lincRNA | 641 | 564 | (87.99%) | 38 | (5.93%) |
| K562 | GRO-cap | Enhancer | 44,833 | 20,067 | (44.76%) | 15,178 | (33.85%) |
| K562 | GRO-cap | Promoter | 8,021 | 5,936 | (74.01%) | 1,747 | (21.78%) |
| K562 | GRO-cap | Transcribed | 2,733 | 215 | (7.87%) | 1,141 | (41.75%) |
| K562 | GRO-cap | CTCF | 15,548 | 1,182 | (7.60%) | 4,618 | (29.70%) |
| K562 | GRO-cap | Repressed | 2,325 | 99 | (4.26%) | 565 | (24.30%) |
| K562 | GRO-cap | Bidirectional | 6,694 | 4,090 | (61.1%) | 1,687 | (25.20%) |
| K562 | GRO-cap | No state | 13,726 | 2,059 | (15.00%) | 4,480 | (32.64%) |
| K562 | GRO-cap | mRNA | 27,528 | 25,882 | (94.02%) | 961 | (3.49%) |
| K562 | GRO-cap | miRNA | 185 | 139 | (75.14%) | 21 | (11.35%) |
| K562 | GRO-cap | lincRNA | 789 | 663 | (84.03%) | 51 | (6.46%) |
| K562 | GRO-seq | Enhancer | 44,833 | 5,872 | (13.10%) | 21,296 | (47.50%) |
| K562 | GRO-seq | Promoter | 8,021 | 2,899 | (36.14%) | 3,809 | (47.49%) |
| K562 | GRO-seq | Transcribed | 2,733 | 292 | (10.68%) | 2,055 | (75.19%) |
| K562 | GRO-seq | CTCF | 15,548 | 451 | (2.90%) | 5,193 | (33.40%) |
| K562 | GRO-seq | Repressed | 2,325 | 23 | (0.99%) | 372 | (16.00%) |
| K562 | GRO-seq | Bidirectional | 6,694 | 1,475 | (22.03%) | 2,956 | (44.16%) |
| K562 | GRO-seq | No state | 13,726 | 893 | (6.51%) | 6,147 | (44.78%) |
| K562 | GRO-seq | mRNA | 27,528 | 21,696 | (78.81%) | 3,456 | (12.55%) |
| K562 | GRO-seq | miRNA | 185 | 108 | (58.38%) | 45 | (24.32%) |
| K562 | GRO-seq | lincRNA | 789 | 493 | (62.48%) | 115 | (14.58%) |
| K562 | PRO-seq | Enhancer | 44,833 | 8,740 | (19.49%) | 24,872 | (55.48%) |
| K562 | PRO-seq | Promoter | 8,021 | 3,697 | (46.09%) | 3,534 | (44.06%) |
| K562 | PRO-seq | Transcribed | 2,733 | 535 | (19.58%) | 2,158 | (78.96%) |
| K562 | PRO-seq | CTCF | 15,548 | 1,283 | (8.25%) | 7,022 | (45.16%) |
| K562 | PRO-seq | Repressed | 2,325 | 73 | (3.14%) | 740 | (31.83%) |
| K562 | PRO-seq | Bidirectional | 6,694 | 1,932 | (28.86%) | 3,210 | (47.95%) |
| K562 | PRO-seq | No state | 13,726 | 1,586 | (11.55%) | 7,885 | (57.45%) |
| K562 | PRO-seq | mRNA | 27,528 | 23,183 | (84.22%) | 976 | (3.55%) |
| K562 | PRO-seq | miRNA | 185 | 121 | (65.41%) | 15 | (8.11%) |
| K562 | PRO-seq | lincRNA | 789 | 534 | (67.68%) | 73 | (9.25%) |
