# Supplementary material for: Bidirectional transcription initiation marks accessible chromatin and is not specific to enhancers
Source: Genome Biol. 2017 Dec 28;18:242. doi: 10.1186/s13059-017-1379-8 (PMC5747114; doi:10.1186/s13059-017-1379-8)
Supplement: Supplementary file 3 — Counts of bidirectionally and unidirectionally transcribed DHSs defined as 250 bp around the midpoint across different chromatin state regions and gene annotations (mRNA/miRNA/lincRNA) as measured by CAGE across cell types and subcellular fractions. (DOC 343 kb) [file 13059_2017_1379_MOESM3_ESM.doc]

**Table S2**: Counts of bidirectionally and unidirectionally transcribed DHSs defined as ± 250 bp around the midpoint across different chromatin state regions and gene annotations (mRNA/miRNA/lincRNA) as measured by CAGE across cell types and subcellular fractions.

| Cell | Sample | Feature | Total | Bidirectional | Percent | Unidirectional | Percent |
| --- | --- | --- | --- | --- | --- | --- | --- |
| Gm12878 | Whole cell, poly(A)^+^ | Enhancer | 22,846 | 1,001 | (4.38%) | 6,073 | (26.58%) |
| Gm12878 | Whole cell, poly(A)^+^ | Promoter | 10,566 | 1,800 | (17.04%) | 4,713 | (44.61%) |
| Gm12878 | Whole cell, poly(A)^+^ | Transcribed | 1,106 | 38 | (3.44%) | 412 | (37.25%) |
| Gm12878 | Whole cell, poly(A)^+^ | CTCF | 10,779 | 141 | (1.31%) | 1,759 | (16.32%) |
| Gm12878 | Whole cell, poly(A)^+^ | Repressed | 113 | 3 | (2.65%) | 13 | (11.50%) |
| Gm12878 | Whole cell, poly(A)^+^ | Bidirectional | 7,064 | 836 | (11.83%) | 2,469 | (34.95%) |
| Gm12878 | Whole cell, poly(A)^+^ | No state | 5,423 | 189 | (3.49%) | 1,188 | (21.91%) |
| Gm12878 | Whole cell, poly(A)^+^ | mRNA | 25,074 | 20,053 | (79.98%) | 4,520 | (18.03%) |
| Gm12878 | Whole cell, poly(A)^+^ | miRNA | 135 | 84 | (62.22%) | 22 | (16.30%) |
| Gm12878 | Whole cell, poly(A)^+^ | lincRNA | 641 | 409 | (63.81%) | 114 | (17.78%) |
| Gm12878 | Cytosol, poly(A)^-^ | Enhancer | 22,846 | 151 | (0.66%) | 3,195 | (13.98%) |
| Gm12878 | Cytosol, poly(A)^-^ | Promoter | 10,566 | 363 | (3.44%) | 2,906 | (27.50%) |
| Gm12878 | Cytosol, poly(A)^-^ | Transcribed | 1,106 | 11 | (0.99%) | 187 | (16.91%) |
| Gm12878 | Cytosol, poly(A)^-^ | CTCF | 10,779 | 141 | (1.31%) | 1,590 | (14.75%) |
| Gm12878 | Cytosol, poly(A)^-^ | Repressed | 113 | 3 | (2.65%) | 13 | (11.50%) |
| Gm12878 | Cytosol, poly(A)^-^ | Bidirectional | 7,064 | 158 | (2.24%) | 1,445 | (20.46%) |
| Gm12878 | Cytosol, poly(A)^-^ | No state | 5,423 | 69 | (1.27%) | 702 | (12.94%) |
| Gm12878 | Cytosol, poly(A)^-^ | mRNA | 25,074 | 16,631 | (66.33%) | 6,560 | (26.16%) |
| Gm12878 | Cytosol, poly(A)^-^ | miRNA | 135 | 63 | (46.67%) | 29 | (21.48%) |
| Gm12878 | Cytosol, poly(A)^-^ | lincRNA | 641 | 333 | (51.95%) | 94 | (14.66%) |
| Gm12878 | Cytosol, poly(A)^+^ | Enhancer | 22,846 | 613 | (2.68%) | 4,529 | (19.82%) |
| Gm12878 | Cytosol, poly(A)^+^ | Promoter | 10,566 | 1,287 | (12.18%) | 4,096 | (38.77%) |
| Gm12878 | Cytosol, poly(A)^+^ | Transcribed | 1,106 | 44 | (3.98%) | 214 | (19.35%) |
| Gm12878 | Cytosol, poly(A)^+^ | CTCF | 10,779 | 106 | (0.98%) | 1,226 | (11.37%) |
| Gm12878 | Cytosol, poly(A)^+^ | Repressed | 113 | 2 | (1.77%) | 13 | (11.50%) |
| Gm12878 | Cytosol, poly(A)^+^ | Bidirectional | 7,064 | 569 | (8.05%) | 2,081 | (29.46%) |
| Gm12878 | Cytosol, poly(A)^+^ | No state | 5,423 | 132 | (2.43%) | 799 | (14.73%) |
| Gm12878 | Cytosol, poly(A)^+^ | mRNA | 25,074 | 19,238 | (76.72%) | 5,245 | (20.92%) |
| Gm12878 | Cytosol, poly(A)^+^ | miRNA | 135 | 80 | (59.26%) | 25 | (18.52%) |
| Gm12878 | Cytosol, poly(A)^+^ | lincRNA | 641 | 394 | (61.47%) | 110 | (17.16%) |
| Gm12878 | Nucleus, poly(A)^-^ | Enhancer | 22,846 | 10 | (0.04%) | 689 | (3.02%) |
| Gm12878 | Nucleus, poly(A)^-^ | Promoter | 10,566 | 46 | (0.44%) | 1,130 | (10.69%) |
| Gm12878 | Nucleus, poly(A)^-^ | Transcribed | 1,106 | 1 | (0.09%) | 26 | (2.35%) |
| Gm12878 | Nucleus, poly(A)^-^ | CTCF | 10,779 | 2 | (0.02%) | 134 | (1.24%) |
| Gm12878 | Nucleus, poly(A)^-^ | Repressed | 113 | 2 | (1.77%) | 1 | (0.88%) |
| Gm12878 | Nucleus, poly(A)^-^ | Bidirectional | 7,064 | 16 | (0.23%) | 455 | (6.44%) |
| Gm12878 | Nucleus, poly(A)^-^ | No state | 5,423 | 20 | (0.37%) | 111 | (2.05%) |
| Gm12878 | Nucleus, poly(A)^-^ | mRNA | 25,074 | 4,214 | (16.81%) | 12,587 | (50.20%) |
| Gm12878 | Nucleus, poly(A)^-^ | miRNA | 135 | 14 | (10.37%) | 43 | (31.85%) |
| Gm12878 | Nucleus, poly(A)^-^ | lincRNA | 641 | 83 | (12.95%) | 111 | (17.32%) |
| Gm12878 | Nucleus, poly(A)^+^ | Enhancer | 22,846 | 1,981 | (8.67%) | 7,773 | (34.02%) |
| Gm12878 | Nucleus, poly(A)^+^ | Promoter | 10,566 | 2,873 | (27.19%) | 4,903 | (46.40%) |
| Gm12878 | Nucleus, poly(A)^+^ | Transcribed | 1,106 | 79 | (7.14%) | 502 | (45.39%) |
| Gm12878 | Nucleus, poly(A)^+^ | CTCF | 10,779 | 237 | (2.20%) | 2,393 | (22.20%) |
| Gm12878 | Nucleus, poly(A)^+^ | Repressed | 113 | 2 | (1.77%) | 22 | (19.47%) |
| Gm12878 | Nucleus, poly(A)^+^ | Bidirectional | 7,064 | 1,390 | (19.68%) | 2,683 | (37.98%) |
| Gm12878 | Nucleus, poly(A)^+^ | No state | 5,423 | 319 | (5.88%) | 1,549 | (28.56%) |
| Gm12878 | Nucleus, poly(A)^+^ | mRNA | 25,074 | 21,166 | (84.41%) | 3,520 | (14.04%) |
| Gm12878 | Nucleus, poly(A)^+^ | miRNA | 135 | 94 | (69.63%) | 20 | (14.81%) |
| Gm12878 | Nucleus, poly(A)^+^ | lincRNA | 641 | 437 | (68.17%) | 105 | (16.38%) |
| Gm12878 | All | Enhancer | 22,846 | 3,303 | (14.46%) | 9,065 | (39.68%) |
| Gm12878 | All | Promoter | 10,566 | 4,105 | (38.85%) | 4,487 | (42.47%) |
| Gm12878 | All | Transcribed | 1,106 | 137 | (12.39%) | 552 | (49.91%) |
| Gm12878 | All | CTCF | 10,779 | 701 | (6.50%) | 3,450 | (32.01%) |
| Gm12878 | All | Repressed | 113 | 7 | (6.19%) | 37 | (32.74%) |
| Gm12878 | All | Bidirectional | 7,064 | 2,141 | (30.31%) | 2,743 | (38.83%) |
| Gm12878 | All | No state | 5,423 | 561 | (10.34%) | 1,895 | (34.94%) |
| Gm12878 | All | mRNA | 25,074 | 23,870 | (95.20%) | 1,087 | (4.34%) |
| Gm12878 | All | miRNA | 135 | 111 | (82.22%) | 9 | (6.67%) |
| Gm12878 | All | lincRNA | 641 | 512 | (79.88%) | 74 | (11.54%) |
| Hepg2 | Whole cell, poly(A)^+^ | Enhancer | 14,634 | 568 | (3.88%) | 4,130 | (28.22%) |
| Hepg2 | Whole cell, poly(A)^+^ | Promoter | 11,711 | 1,131 | (9.66%) | 4,523 | (38.62%) |
| Hepg2 | Whole cell, poly(A)^+^ | Transcribed | 1,040 | 71 | (6.83%) | 350 | (33.65%) |
| Hepg2 | Whole cell, poly(A)^+^ | CTCF | 7,279 | 161 | (2.21%) | 1,355 | (18.62%) |
| Hepg2 | Whole cell, poly(A)^+^ | Repressed | 202 | 8 | (3.96%) | 41 | (20.30%) |
| Hepg2 | Whole cell, poly(A)^+^ | Bidirectional | 3,657 | 275 | (7.52%) | 1,288 | (35.22%) |
| Hepg2 | Whole cell, poly(A)^+^ | No state | 6,856 | 207 | (3.02%) | 1,240 | (18.09%) |
| Hepg2 | Whole cell, poly(A)^+^ | mRNA | 29,432 | 20,638 | (70.12%) | 7,795 | (26.48%) |
| Hepg2 | Whole cell, poly(A)^+^ | miRNA | 150 | 84 | (56.00%) | 29 | (19.33%) |
| Hepg2 | Whole cell, poly(A)^+^ | lincRNA | 702 | 428 | (60.97%) | 132 | (18.80%) |
| Hepg2 | Cytosol, poly(A)^-^ | Enhancer | 14,634 | 340 | (2.32%) | 3,112 | (21.27%) |
| Hepg2 | Cytosol, poly(A)^-^ | Promoter | 11,711 | 737 | (6.29%) | 3,713 | (31.71%) |
| Hepg2 | Cytosol, poly(A)^-^ | Transcribed | 1,040 | 47 | (4.52%) | 232 | (22.31%) |
| Hepg2 | Cytosol, poly(A)^-^ | CTCF | 7,279 | 145 | (1.99%) | 1,420 | (19.51%) |
| Hepg2 | Cytosol, poly(A)^-^ | Repressed | 202 | 11 | (5.45%) | 44 | (21.78%) |
| Hepg2 | Cytosol, poly(A)^-^ | Bidirectional | 3,657 | 207 | (5.66%) | 1,038 | (28.38%) |
| Hepg2 | Cytosol, poly(A)^-^ | No state | 6,856 | 172 | (2.51%) | 883 | (12.88%) |
| Hepg2 | Cytosol, poly(A)^-^ | mRNA | 29,432 | 20,040 | (68.09%) | 7,929 | (26.94%) |
| Hepg2 | Cytosol, poly(A)^-^ | miRNA | 150 | 89 | (59.33%) | 19 | (12.67%) |
| Hepg2 | Cytosol, poly(A)^-^ | lincRNA | 702 | 382 | (54.42%) | 141 | (20.09%) |
| Hepg2 | Cytosol, poly(A)^+^ | Enhancer | 14,634 | 376 | (2.57%) | 2,945 | (20.12%) |
| Hepg2 | Cytosol, poly(A)^+^ | Promoter | 11,711 | 922 | (7.87%) | 3,788 | (32.35%) |
| Hepg2 | Cytosol, poly(A)^+^ | Transcribed | 1,040 | 51 | (4.90%) | 209 | (20.10%) |
| Hepg2 | Cytosol, poly(A)^+^ | CTCF | 7,279 | 177 | (2.43%) | 1,094 | (15.03%) |
| Hepg2 | Cytosol, poly(A)^+^ | Repressed | 202 | 10 | (4.95%) | 43 | (21.29%) |
| Hepg2 | Cytosol, poly(A)^+^ | Bidirectional | 3,657 | 234 | (6.40%) | 1,034 | (28.27%) |
| Hepg2 | Cytosol, poly(A)^+^ | No state | 6,856 | 153 | (2.23%) | 756 | (11.03%) |
| Hepg2 | Cytosol, poly(A)^+^ | mRNA | 29,432 | 20,798 | (70.66%) | 7,677 | (26.08%) |
| Hepg2 | Cytosol, poly(A)^+^ | miRNA | 150 | 79 | (52.67%) | 29 | (19.33%) |
| Hepg2 | Cytosol, poly(A)^+^ | lincRNA | 702 | 415 | (59.12%) | 144 | (20.51%) |
| Hepg2 | Nucleus, poly(A)^-^ | Enhancer | 14,634 | 513 | (3.51%) | 3,923 | (26.81%) |
| Hepg2 | Nucleus, poly(A)^-^ | Promoter | 11,711 | 421 | (3.59%) | 3,232 | (27.60%) |
| Hepg2 | Nucleus, poly(A)^-^ | Transcribed | 1,040 | 99 | (9.52%) | 462 | (44.42%) |
| Hepg2 | Nucleus, poly(A)^-^ | CTCF | 7,279 | 153 | (2.10%) | 1,470 | (20.20%) |
| Hepg2 | Nucleus, poly(A)^-^ | Repressed | 202 | 9 | (4.46%) | 35 | (17.33%) |
| Hepg2 | Nucleus, poly(A)^-^ | Bidirectional | 3,657 | 116 | (3.17%) | 911 | (24.91%) |
| Hepg2 | Nucleus, poly(A)^-^ | No state | 6,856 | 313 | (4.57%) | 1,912 | (27.89%) |
| Hepg2 | Nucleus, poly(A)^-^ | mRNA | 29,432 | 8,640 | (29.36%) | 15,246 | (51.80%) |
| Hepg2 | Nucleus, poly(A)^-^ | miRNA | 150 | 35 | (23.33%) | 41 | (27.33%) |
| Hepg2 | Nucleus, poly(A)^-^ | lincRNA | 702 | 204 | (29.06%) | 142 | (20.23%) |
| Hepg2 | Nucleus, poly(A)^+^ | Enhancer | 14,634 | 1,020 | (6.97%) | 5,423 | (37.06%) |
| Hepg2 | Nucleus, poly(A)^+^ | Promoter | 11,711 | 1,797 | (15.34%) | 5,116 | (43.69%) |
| Hepg2 | Nucleus, poly(A)^+^ | Transcribed | 1,040 | 72 | (6.92%) | 502 | (48.27%) |
| Hepg2 | Nucleus, poly(A)^+^ | CTCF | 7,279 | 177 | (2.43%) | 1,703 | (23.40%) |
| Hepg2 | Nucleus, poly(A)^+^ | Repressed | 202 | 6 | (2.97%) | 36 | (17.82%) |
| Hepg2 | Nucleus, poly(A)^+^ | Bidirectional | 3,657 | 496 | (13.56%) | 1,428 | (39.05%) |
| Hepg2 | Nucleus, poly(A)^+^ | No state | 6,856 | 244 | (3.56%) | 1,704 | (24.85%) |
| Hepg2 | Nucleus, poly(A)^+^ | mRNA | 29,432 | 21,793 | (74.05%) | 6,826 | (23.19%) |
| Hepg2 | Nucleus, poly(A)^+^ | miRNA | 150 | 92 | (61.33%) | 31 | (20.67%) |
| Hepg2 | Nucleus, poly(A)^+^ | lincRNA | 702 | 453 | (64.53%) | 120 | (17.09%) |
| Hepg2 | All | Enhancer | 14,634 | 2,981 | (20.37%) | 6,605 | (45.13%) |
| Hepg2 | All | Promoter | 11,711 | 3,869 | (33.04%) | 5,155 | (44.02%) |
| Hepg2 | All | Transcribed | 1,040 | 269 | (25.87%) | 532 | (51.15%) |
| Hepg2 | All | CTCF | 7,279 | 926 | (12.72%) | 2,879 | (39.55%) |
| Hepg2 | All | Repressed | 202 | 35 | (17.33%) | 69 | (34.16%) |
| Hepg2 | All | Bidirectional | 3,657 | 1,098 | (30.02%) | 1,557 | (42.58%) |
| Hepg2 | All | No state | 6,856 | 809 | (11.80%) | 2,748 | (40.08%) |
| Hepg2 | All | mRNA | 29,432 | 27,651 | (93.95%) | 1,615 | (5.49%) |
| Hepg2 | All | miRNA | 150 | 127 | (84.67%) | 9 | (6.00%) |
| Hepg2 | All | lincRNA | 702 | 612 | (87.18%) | 53 | (7.55%) |
| Huvec | Whole cell, poly(A)^+^ | Enhancer | 28,664 | 747 | (2.61%) | 6,726 | (23.46%) |
| Huvec | Whole cell, poly(A)^+^ | Promoter | 3,586 | 327 | (9.12%) | 1,290 | (35.97%) |
| Huvec | Whole cell, poly(A)^+^ | Transcribed | 941 | 33 | (3.51%) | 254 | (26.99%) |
| Huvec | Whole cell, poly(A)^+^ | CTCF | 14,694 | 97 | (0.66%) | 1,573 | (10.71%) |
| Huvec | Whole cell, poly(A)^+^ | Repressed | 420 | 1 | (0.24%) | 20 | (4.76%) |
| Huvec | Whole cell, poly(A)^+^ | Bidirectional | 6,268 | 225 | (3.59%) | 1,494 | (23.84%) |
| Hepg2 | Whole cell, poly(A)^+^ | No state | 18,086 | 110 | (0.61%) | 1,848 | (10.22%) |
| Huvec | Whole cell, poly(A)^+^ | mRNA | 22,942 | 14,777 | (64.41%) | 7,594 | (33.10%) |
| Huvec | Whole cell, poly(A)^+^ | miRNA | 110 | 51 | (46.36%) | 25 | (22.73%) |
| Huvec | Whole cell, poly(A)^+^ | lincRNA | 616 | 319 | (51.79%) | 145 | (23.54%) |
| Huvec | Cytosol, poly(A)^-^ | Enhancer | 28,664 | 1,285 | (4.48%) | 7,789 | (27.17%) |
| Huvec | Cytosol, poly(A)^-^ | Promoter | 3,586 | 382 | (10.65%) | 1,320 | (36.81%) |
| Huvec | Cytosol, poly(A)^-^ | Transcribed | 941 | 45 | (4.78%) | 289 | (30.71%) |
| Huvec | Cytosol, poly(A)^-^ | CTCF | 14,694 | 138 | (0.94%) | 1,812 | (12.33%) |
| Huvec | Cytosol, poly(A)^-^ | Repressed | 420 | 2 | (0.48%) | 30 | (7.14%) |
| Huvec | Cytosol, poly(A)^-^ | Bidirectional | 6,268 | 392 | (6.25%) | 1,815 | (28.96%) |
| Huvec | Cytosol, poly(A)^-^ | No state | 18,086 | 185 | (1.02%) | 2,161 | (11.95%) |
| Huvec | Cytosol, poly(A)^-^ | mRNA | 22,942 | 15,488 | (67.51%) | 6,601 | (28.77%) |
| Huvec | Cytosol, poly(A)^-^ | miRNA | 110 | 57 | (51.82%) | 19 | (17.27%) |
| Huvec | Cytosol, poly(A)^-^ | lincRNA | 616 | 331 | (53.73%) | 96 | (15.58%) |
| Huvec | Cytosol, poly(A)^+^ | Enhancer | 28,664 | 573 | (2.0%) | 5,162 | (18.01%) |
| Huvec | Cytosol, poly(A)^+^ | Promoter | 3,586 | 298 | (8.31%) | 1,144 | (31.90%) |
| Huvec | Cytosol, poly(A)^+^ | Transcribed | 941 | 31 | (3.29%) | 188 | (19.98%) |
| Huvec | Cytosol, poly(A)^+^ | CTCF | 14,694 | 170 | (1.16%) | 1,492 | (10.15%) |
| Huvec | Cytosol, poly(A)^+^ | Repressed | 420 | 0 | (0.00%) | 25 | (5.95%) |
| Huvec | Cytosol, poly(A)^+^ | Bidirectional | 6,268 | 156 | (2.49%) | 1,214 | (19.37%) |
| Huvec | Cytosol, poly(A)^+^ | No state | 18,086 | 118 | (0.65%) | 1,391 | (7.69%) |
| Huvec | Cytosol, poly(A)^+^ | mRNA | 22,942 | 15,702 | (68.44%) | 6,635 | (28.92%) |
| Huvec | Cytosol, poly(A)^+^ | miRNA | 110 | 53 | (48.18%) | 19 | (17.27%) |
| Huvec | Cytosol, poly(A)^+^ | lincRNA | 616 | 308 | (50.00%) | 142 | (23.05%) |
| Huvec | Nucleus, poly(A)^-^ | Enhancer | 28,664 | 2,956 | (10.31%) | 10,419 | (36.35%) |
| Huvec | Nucleus, poly(A)^-^ | Promoter | 3,586 | 794 | (22.14%) | 1,410 | (39.32%) |
| Huvec | Nucleus, poly(A)^-^ | Transcribed | 941 | 45 | (4.78%) | 397 | (42.19%) |
| Huvec | Nucleus, poly(A)^-^ | CTCF | 14,694 | 101 | (0.69%) | 1,791 | (12.19%) |
| Huvec | Nucleus, poly(A)^-^ | Repressed | 420 | 0 | (0.00%) | 22 | (5.24%) |
| Huvec | Nucleus, poly(A)^-^ | Bidirectional | 6,268 | 900 | (14.36%) | 2,184 | (34.84%) |
| Huvec | Nucleus, poly(A)^-^ | No state | 18,086 | 240 | (1.33%) | 3,010 | (16.64%) |
| Huvec | Nucleus, poly(A)^-^ | mRNA | 22,942 | 15,617 | (68.07%) | 6,730 | (29.33%) |
| Huvec | Nucleus, poly(A)^-^ | miRNA | 110 | 53 | (48.18%) | 24 | (21.82%) |
| Huvec | Nucleus, poly(A)^-^ | lincRNA | 616 | 365 | (59.25%) | 107 | (17.37%) |
| Huvec | Nucleus, poly(A)^+^ | Enhancer | 28,664 | 1,972 | (6.88%) | 11,652 | (40.65%) |
| Huvec | Nucleus, poly(A)^+^ | Promoter | 3,586 | 553 | (15.42%) | 1,636 | (45.62%) |
| Huvec | Nucleus, poly(A)^+^ | Transcribed | 941 | 63 | (6.70%) | 597 | (63.44%) |
| Huvec | Nucleus, poly(A)^+^ | CTCF | 14,694 | 286 | (1.95%) | 3,293 | (22.41%) |
| Huvec | Nucleus, poly(A)^+^ | Repressed | 420 | 3 | (0.71%) | 46 | (10.95%) |
| Huvec | Nucleus, poly(A)^+^ | Bidirectional | 6,268 | 537 | (8.57%) | 2,284 | (36.44%) |
| Huvec | Nucleus, poly(A)^+^ | No state | 18,086 | 330 | (1.82%) | 4,531 | (25.05%) |
| Huvec | Nucleus, poly(A)^+^ | mRNA | 22,942 | 15,739 | (68.6%) | 6,734 | (29.35%) |
| Huvec | Nucleus, poly(A)^+^ | miRNA | 110 | 56 | (50.91%) | 29 | (26.36%) |
| Huvec | Nucleus, poly(A)^+^ | lincRNA | 616 | 346 | (56.17%) | 124 | (20.13%) |
| Huvec | All | Enhancer | 28,664 | 6,250 | (21.80%) | 13,095 | (45.68%) |
| Huvec | All | Promoter | 3,586 | 1,406 | (39.21%) | 1,467 | (40.91%) |
| Huvec | All | Transcribed | 941 | 154 | (16.37%) | 576 | (61.21%) |
| Huvec | All | CTCF | 14,694 | 808 | (5.50%) | 4,525 | (30.79%) |
| Huvec | All | Repressed | 420 | 10 | (2.38%) | 81 | (19.29%) |
| Huvec | All | Bidirectional | 6,268 | 1,648 | (26.29%) | 2,598 | (41.45%) |
| Huvec | All | No state | 18,086 | 957 | (5.29%) | 5,997 | (33.16%) |
| Huvec | All | mRNA | 22,942 | 21,080 | (91.88%) | 1,720 | (7.50%) |
| Huvec | All | miRNA | 110 | 83 | (75.45%) | 15 | (13.64%) |
| Huvec | All | lincRNA | 616 | 480 | (77.92%) | 73 | (11.85%) |
| K562 | Whole cell, poly(A)^+^ | Enhancer | 44,833 | 606 | (1.35%) | 7,444 | (16.60%) |
| K562 | Whole cell, poly(A)^+^ | Promoter | 8,021 | 702 | (8.75%) | 3,038 | (37.88%) |
| K562 | Whole cell, poly(A)^+^ | Transcribed | 2,733 | 65 | (2.38%) | 747 | (27.33%) |
| K562 | Whole cell, poly(A)^+^ | CTCF | 15,548 | 100 | (0.64%) | 1,751 | (11.26%) |
| K562 | Whole cell, poly(A)^+^ | Repressed | 2,325 | 7 | (0.30%) | 132 | (5.68%) |
| K562 | Whole cell, poly(A)^+^ | Bidirectional | 6,694 | 260 | (3.88%) | 1,433 | (21.41%) |
| K562 | Whole cell, poly(A)^+^ | No state | 13,726 | 189 | (1.38%) | 2,172 | (15.82%) |
| K562 | Whole cell, poly(A)^+^ | mRNA | 27,528 | 18,514 | (67.26%) | 7,912 | (28.74%) |
| K562 | Whole cell, poly(A)^+^ | miRNA | 185 | 76 | (41.08%) | 47 | (25.41%) |
| K562 | Whole cell, poly(A)^+^ | lincRNA | 789 | 388 | (49.18%) | 177 | (22.43%) |
| K562 | Cytosol, poly(A)^-^ | Enhancer | 44,833 | 82 | (0.18%) | 2,488 | (5.55%) |
| K562 | Cytosol, poly(A)^-^ | Promoter | 8,021 | 86 | (1.07%) | 1,328 | (16.56%) |
| K562 | Cytosol, poly(A)^-^ | Transcribed | 2,733 | 29 | (1.06%) | 295 | (10.79%) |
| K562 | Cytosol, poly(A)^-^ | CTCF | 15,548 | 23 | (0.15%) | 523 | (3.36%) |
| K562 | Cytosol, poly(A)^-^ | Repressed | 2,325 | 3 | (0.13%) | 40 | (1.72%) |
| K562 | Cytosol, poly(A)^-^ | Bidirectional | 6,694 | 18 | (0.27%) | 465 | (6.95%) |
| K562 | Cytosol, poly(A)^-^ | No state | 13,726 | 88 | (0.64%) | 846 | (6.16%) |
| K562 | Cytosol, poly(A)^-^ | mRNA | 27,528 | 7,371 | (26.78%) | 16,660 | (60.52%) |
| K562 | Cytosol, poly(A)^-^ | miRNA | 185 | 29 | (15.68%) | 48 | (25.95%) |
| K562 | Cytosol, poly(A)^-^ | lincRNA | 789 | 172 | (21.80%) | 172 | (21.80%) |
| K562 | Cytosol, poly(A)^+^ | Enhancer | 44,833 | 1,071 | (2.39%) | 8,806 | (19.64%) |
| K562 | Cytosol, poly(A)^+^ | Promoter | 8,021 | 1,268 | (15.81%) | 3,048 | (38.00%) |
| K562 | Cytosol, poly(A)^+^ | Transcribed | 2,733 | 98 | (3.59%) | 565 | (20.67%) |
| K562 | Cytosol, poly(A)^+^ | CTCF | 15,548 | 368 | (2.37%) | 2,850 | (18.33%) |
| K562 | Cytosol, poly(A)^+^ | Repressed | 2,325 | 24 | (1.03%) | 307 | (13.20%) |
| K562 | Cytosol, poly(A)^+^ | Bidirectional | 6,694 | 450 | (6.72%) | 1,772 | (26.47%) |
| K562 | Cytosol, poly(A)^+^ | No state | 13,726 | 317 | (2.31%) | 2,147 | (15.64%) |
| K562 | Cytosol, poly(A)^+^ | mRNA | 27,528 | 23,193 | (84.25%) | 3,674 | (13.35%) |
| K562 | Cytosol, poly(A)^+^ | miRNA | 185 | 104 | (56.22%) | 35 | (18.92%) |
| K562 | Cytosol, poly(A)^+^ | lincRNA | 789 | 484 | (61.34%) | 151 | (19.14%) |
| K562 | Nucleus, poly(A)^-^ | Enhancer | 44,833 | 599 | (1.34%) | 9,908 | (22.10%) |
| K562 | Nucleus, poly(A)^-^ | Promoter | 8,021 | 349 | (4.35%) | 2,845 | (35.47%) |
| K562 | Nucleus, poly(A)^-^ | Transcribed | 2,733 | 105 | (3.84%) | 1,099 | (40.21%) |
| K562 | Nucleus, poly(A)^-^ | CTCF | 15,548 | 84 | (0.54%) | 2,014 | (12.95%) |
| K562 | Nucleus, poly(A)^-^ | Repressed | 2,325 | 12 | (0.52%) | 182 | (7.83%) |
| K562 | Nucleus, poly(A)^-^ | Bidirectional | 6,694 | 116 | (1.73%) | 1,533 | (22.90%) |
| K562 | Nucleus, poly(A)^-^ | No state | 13,726 | 325 | (2.37%) | 3,539 | (25.78%) |
| K562 | Nucleus, poly(A)^-^ | mRNA | 27,528 | 9,443 | (34.30%) | 14,757 | (53.61%) |
| K562 | Nucleus, poly(A)^-^ | miRNA | 185 | 33 | (17.84%) | 67 | (36.22%) |
| K562 | Nucleus, poly(A)^-^ | lincRNA | 789 | 217 | (27.50%) | 184 | (23.32%) |
| K562 | Nucleus, poly(A)^+^ | Enhancer | 44,833 | 908 | (2.03%) | 10,262 | (22.89%) |
| K562 | Nucleus, poly(A)^+^ | Promoter | 8,021 | 1,098 | (13.69%) | 3,434 | (42.81%) |
| K562 | Nucleus, poly(A)^+^ | Transcribed | 2,733 | 72 | (2.63%) | 1,037 | (37.94%) |
| K562 | Nucleus, poly(A)^+^ | CTCF | 15,548 | 163 | (1.05%) | 2,505 | (16.11%) |
| K562 | Nucleus, poly(A)^+^ | Repressed | 2,325 | 12 | (0.52%) | 192 | (8.26%) |
| K562 | Nucleus, poly(A)^+^ | Bidirectional | 6,694 | 347 | (5.18%) | 1,875 | (28.01%) |
| K562 | Nucleus, poly(A)^+^ | No state | 13,726 | 271 | (1.97%) | 3,026 | (22.05%) |
| K562 | Nucleus, poly(A)^+^ | mRNA | 27,528 | 20,717 | (75.26%) | 6,039 | (21.94%) |
| K562 | Nucleus, poly(A)^+^ | miRNA | 185 | 92 | (49.73%) | 45 | (24.32%) |
| K562 | Nucleus, poly(A)^+^ | lincRNA | 789 | 445 | (56.4%) | 159 | (20.15%) |
| K562 | All | Enhancer | 44,833 | 3628 | (8.09%) | 17,374 | (38.75%) |
| K562 | All | Promoter | 8,021 | 2455 | (30.61%) | 3,667 | (45.72%) |
| K562 | All | Transcribed | 2,733 | 342 | (12.51%) | 1,417 | (51.85%) |
| K562 | All | CTCF | 15,548 | 824 | (5.30%) | 4,783 | (30.76%) |
| K562 | All | Repressed | 2,325 | 61 | (2.62%) | 527 | (22.67%) |
| K562 | All | Bidirectional | 6,694 | 1070 | (15.98%) | 2,674 | (39.95%) |
| K562 | All | No state | 13,726 | 1024 | (7.46%) | 5,053 | (36.81%) |
| K562 | All | mRNA | 27,528 | 25261 | (91.76%) | 2,008 | (7.29%) |
| K562 | All | miRNA | 185 | 129 | (69.73%) | 35 | (18.92%) |
| K562 | All | lincRNA | 789 | 584 | (74.02%) | 109 | (13.81%) |
