# Supplementary material for: Bidirectional transcription initiation marks accessible chromatin and is not specific to enhancers
Source: Genome Biol. 2017 Dec 28;18:242. doi: 10.1186/s13059-017-1379-8 (PMC5747114; doi:10.1186/s13059-017-1379-8)
Supplement: Supplementary file 4 — Number of chromatin state loci and bidirectionally transcribed-defined enhancers whose measure of stable transcription initiation and accessible chromatin is significantly correlated with transcription from putative annotated gene promoter targets as measured by either transcription initiation from the maximally expressed TSS (single TSS) or the sum of all annotated promoters (summed TSSs) associated with that gene. (DOC 51 kb) [file 13059_2017_1379_MOESM4_ESM.doc]

**Table S3**: Number of chromatin state loci and bidirectionally transcribed-defined enhancers whose measure of stable transcription initiation and accessible chromatin is significantly correlated with transcription from putative annotated gene promoter targets as measured by either transcription initiation from the maximally-expressed TSS (single TSS) or the sum of all annotated promoters (summed TSSs) associated with that gene.

|  |  |  | **Stable transcription** | | **Open chromatin** | |
| --- | --- | --- | --- | --- | --- | --- |
| **Genomic annotation** | **Total** | **Targeting** | **Single TSS** | **Summed TSSs** | **Single TSS** | **Summed TSSs** |
| Enhancer | 96,343 | Closest | 6,713 (6.97%) | 8,452 (8.77%) | 10,961 (11.38%) | 11,827 (12.28%) |
| Promoter | 25,507 | Closest | 1,915 (7.51%) | 2,286 (8.96%) | 2,529 (9.91%) | 2,720 (10.66%) |
| CTCF | 27,569 | Closest | 1,587 (5.76%) | 1,873 (6.79%) | 1,232 (4.47%) | 1,295 (4.70%) |
| Repressed | 6,390 | Closest | 358 (5.60%) | 421 (6.59%) | 226 (3.54%) | 237 (3.71%) |
| Bidirectional | 776 | Closest | 53 (6.83%) | 71 (9.15%) | 7 (0.90%) | 4 (0.52%) |
| No state | 66 | Closest | 2 (3.03%) | 3 (4.55%) | 6 (9.09%) | 5 (7.58%) |
| Enhancer | 96,343 | 500 kb | 48,257 (50.09%) | 51,156 (53.10%) | 67,744 (70.32%) | 67,957 (70.54%) |
| Promoter | 25,507 | 500 kb | 12,682 (49.72%) | 13,150 (51.55%) | 15,776 (61.85%) | 15,752 (61.76%) |
| CTCF | 27,569 | 500 kb | 11,913 (43.21%) | 12,665 (45.94%) | 11,096 (40.25%) | 11,099 (40.26%) |
| Repressed | 6,390 | 500 kb | 3,839 (60.08%) | 3,779 (59.14%) | 3,211 (50.25%) | 3,179 (49.65) |
| Bidirectional | 776 | 500 kb | 535 (68.94%) | 534 (68.81%) | 186 (23.97%) | 247 (31.83%) |
| No state | 66 | 500 kb | 31 (46.97%) | 21 (31.82%) | 21 (31.82%) | 22 (33.33%) |
| Enhancer | 96,343 | Domain | 38,801 (40.27%) | 41,403 (42.97%) | 55,983 (58.11%) | 56,138 (58.27%) |
| Promoter | 25,507 | Domain | 9,910 (38.85%) | 10,414 (40.83%) | 12,444 (48.79%) | 12,414 (48.67%) |
| CTCF | 27,569 | Domain | 8,845 (32.08%) | 9,411 (34.14%) | 8,148 (29.55%) | 8,130 (29.49%) |
| Repressed | 6,390 | Domain | 2,751 (43.05%) | 2,750 (43.04%) | 2,307 (36.10%) | 2,337 (36.57%) |
| Bidirectional | 776 | Domain | 408 (52.58%) | 408 (52.58%) | 408 (52.58%) | 408 (52.58%) |
| No state | 66 | Domain | 23 (34.85%) | 17 (25.76%) | 23 (34.85%) | 17 (25.76%) |
| Enhancer | 96,343 | Loop | 1,774 (1.84%) | 2,048 (2.13%) | 2,619 (2.62%) | 2,695 (2.80%) |
| Promoter | 25,507 | Loop | 550 (2.16%) | 631 (2.47%) | 744 (2.92%) | 754 (2.96%) |
| CTCF | 27,569 | Loop | 318 (1.15%) | 339 (1.23%) | 288 (1.04%) | 312 (1.13%) |
| Repressed | 6,390 | Loop | 74 (1.16%) | 74 (1.16%) | 58 (0.91%) | 69 (1.08%) |
| Bidirectional | 776 | Loop | 32 (4.12%) | 38 (4.90%) | 32 (4.12%) | 38 (4.90%) |
| No state | 66 | Loop | 0 (0.00%) | 0 (0.00%) | 0 (0.00%) | 0 (0.00%) |
