# Supplementary material for: Bidirectional transcription initiation marks accessible chromatin and is not specific to enhancers
Source: Genome Biol. 2017 Dec 28;18:242. doi: 10.1186/s13059-017-1379-8 (PMC5747114; doi:10.1186/s13059-017-1379-8)
Supplement: Supplementary file 5 — Counts of single-exonic and multi-exonic transcripts built by Cufflinks and filtered as described in the “Methods” section (‘Transcriptome analysis’) for each annotation class analysed. (DOC 30 kb) [file 13059_2017_1379_MOESM5_ESM.doc]

**Table S4**: Counts of single-exonic and multi-exonic transcripts built by Cufflinks and filtered as described in the Methods section (‘Transcriptome analysis’) for each annotation class analysed.

| Transcription annotation | No. single-exonic transcripts | No. multi-exonic transcripts |
| --- | --- | --- |
| protein-coding | 55 | 136,435 |
| lncRNA | 4 | 14,547 |
| Enhancer | 42 | 694 |
| Promoter | 13 | 206 |
| CTCF | 41 | 258 |
| Repressed | 2 | 138 |
| Bidirectional | 13 | 1,836 |
| No state | 2,958 | 7,032 |
