# Supplementary material for: Bidirectional transcription initiation marks accessible chromatin and is not specific to enhancers
Source: Genome Biol. 2017 Dec 28;18:242. doi: 10.1186/s13059-017-1379-8 (PMC5747114; doi:10.1186/s13059-017-1379-8)

**Table S5**: Primer sequences, coordinates, and reporter construct activities measured in HepG2 cells.


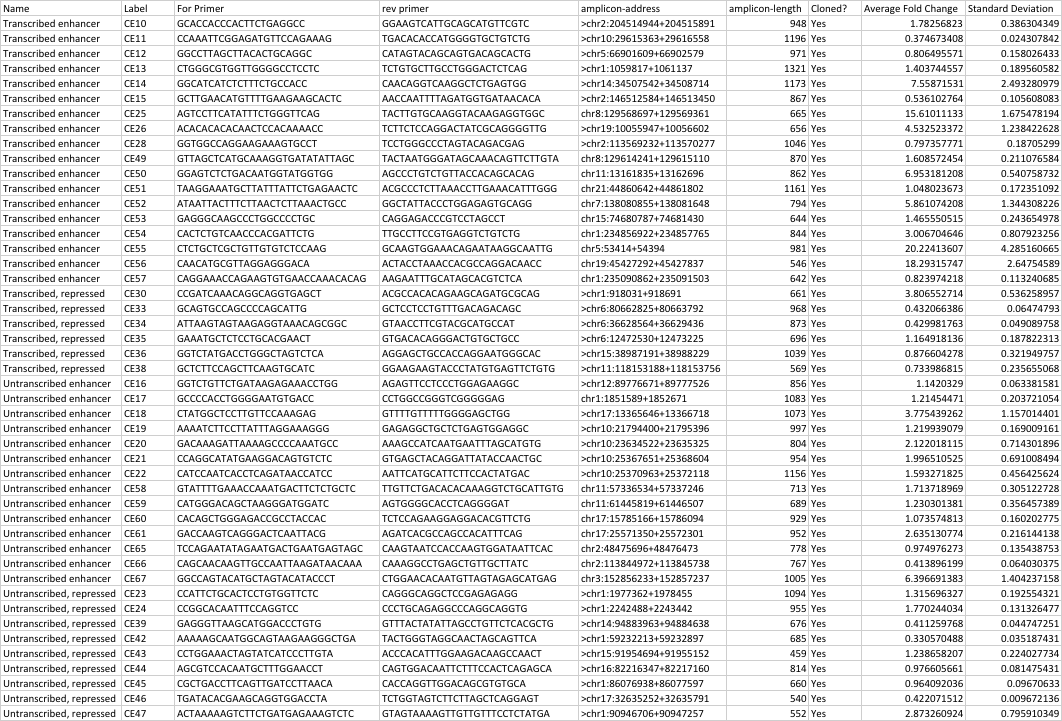

Supplement: Supplementary file 6 — Primer sequences, coordinates, and reporter construct activities measured in HepG2 cells. (DOC 294 kb) [file 13059_2017_1379_MOESM6_ESM.doc]
